# Supplementary material for: Reference ranges and Z-scores for fetal cardiac measurements from two-dimensional echocardiography in Asian population
Source: PLoS One. 2020 Jun 25;15(6):e0233179. doi: 10.1371/journal.pone.0233179 (PMC7316227; doi:10.1371/journal.pone.0233179)
Supplement: S1 Fig — a-m. Centile graphs by estimated gestational age, bi-parietal distance, femur length, abdominal circumference, head circumference. (PDF) [file pone.0233179.s001.pdf]

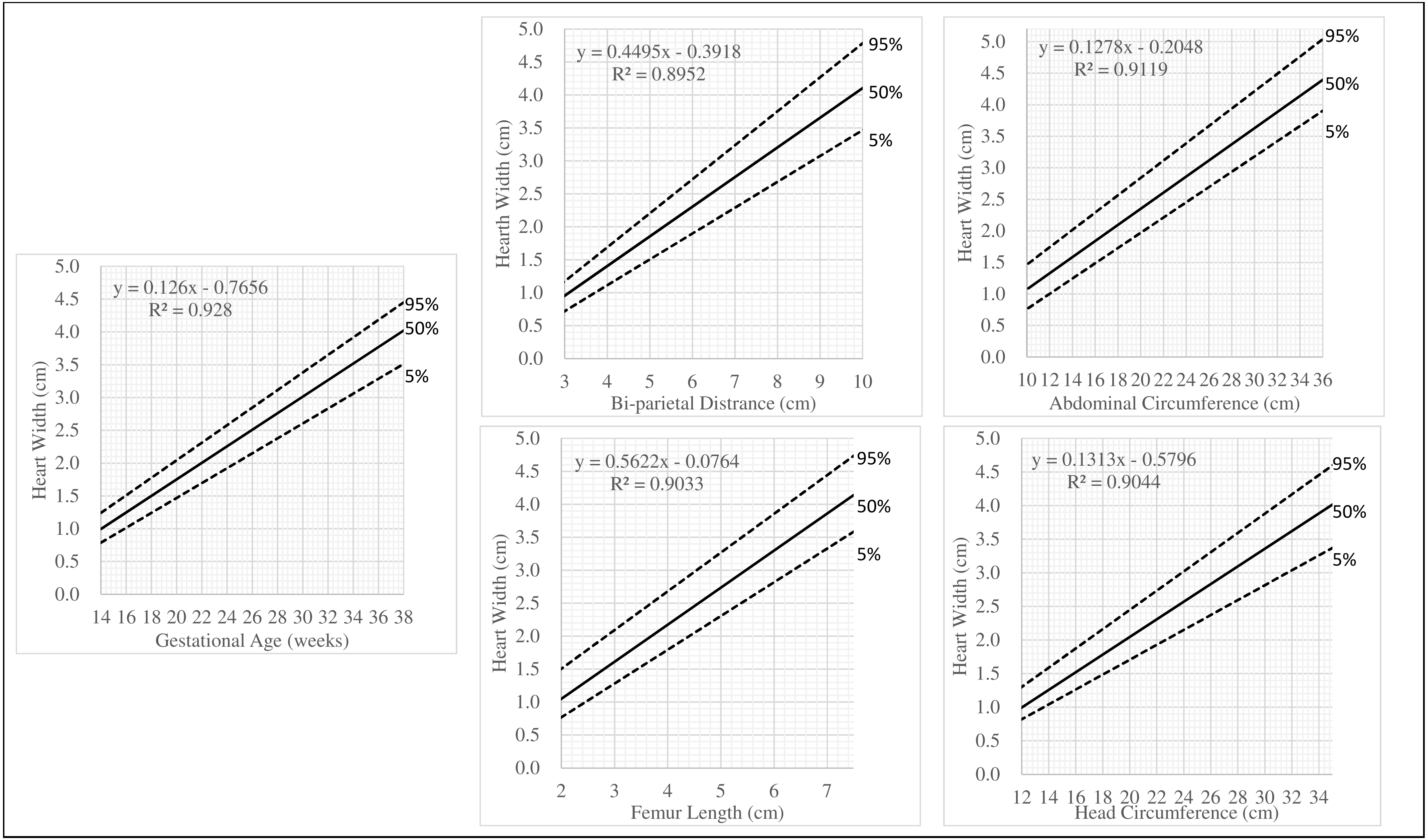

Fig. S1.a. Centile graphs for heart width by estimated gestional age, bi-parietal distance, femur length, abdominal circumference, head circumference.

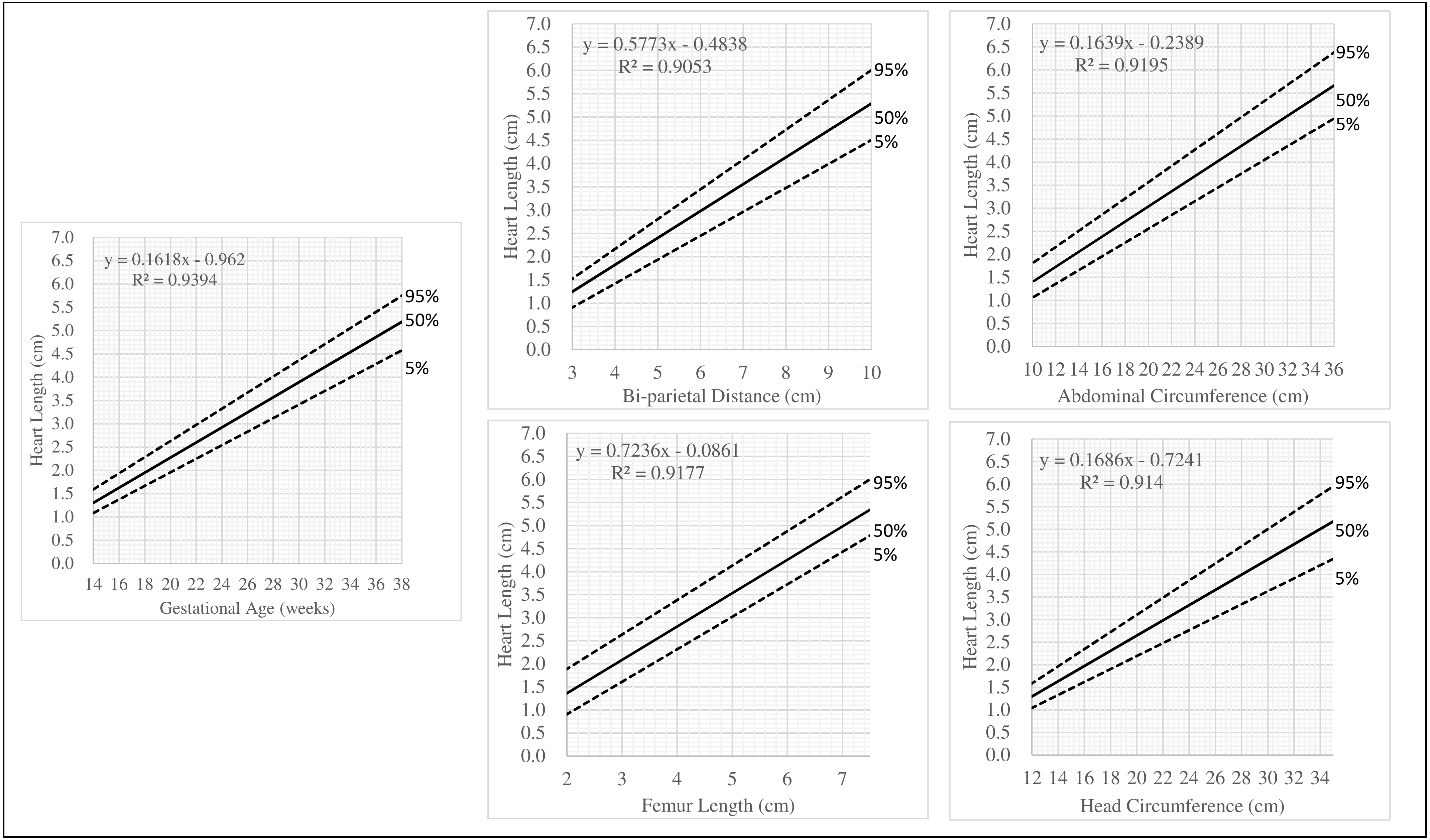

Fig. S1.b. Centile graphs for heart length by estimated gestional age, bi-parietal distance, femur length, abdominal circumference, head circumference.

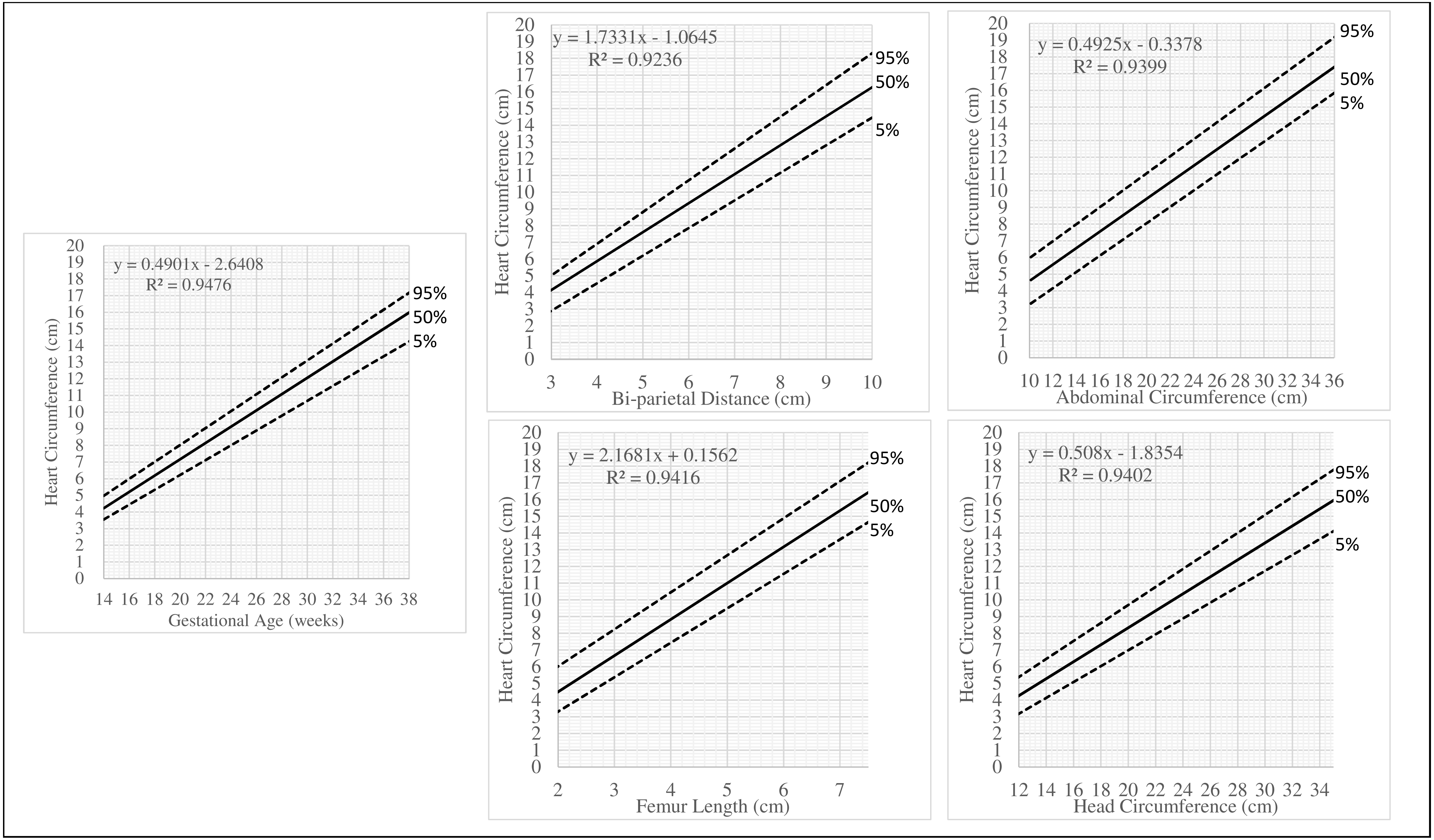

Fig. S1.c. Centile graphs for heart circumference by estimated gestional age, bi-parietal distance, femur length, abdominal circumference, head circumference.

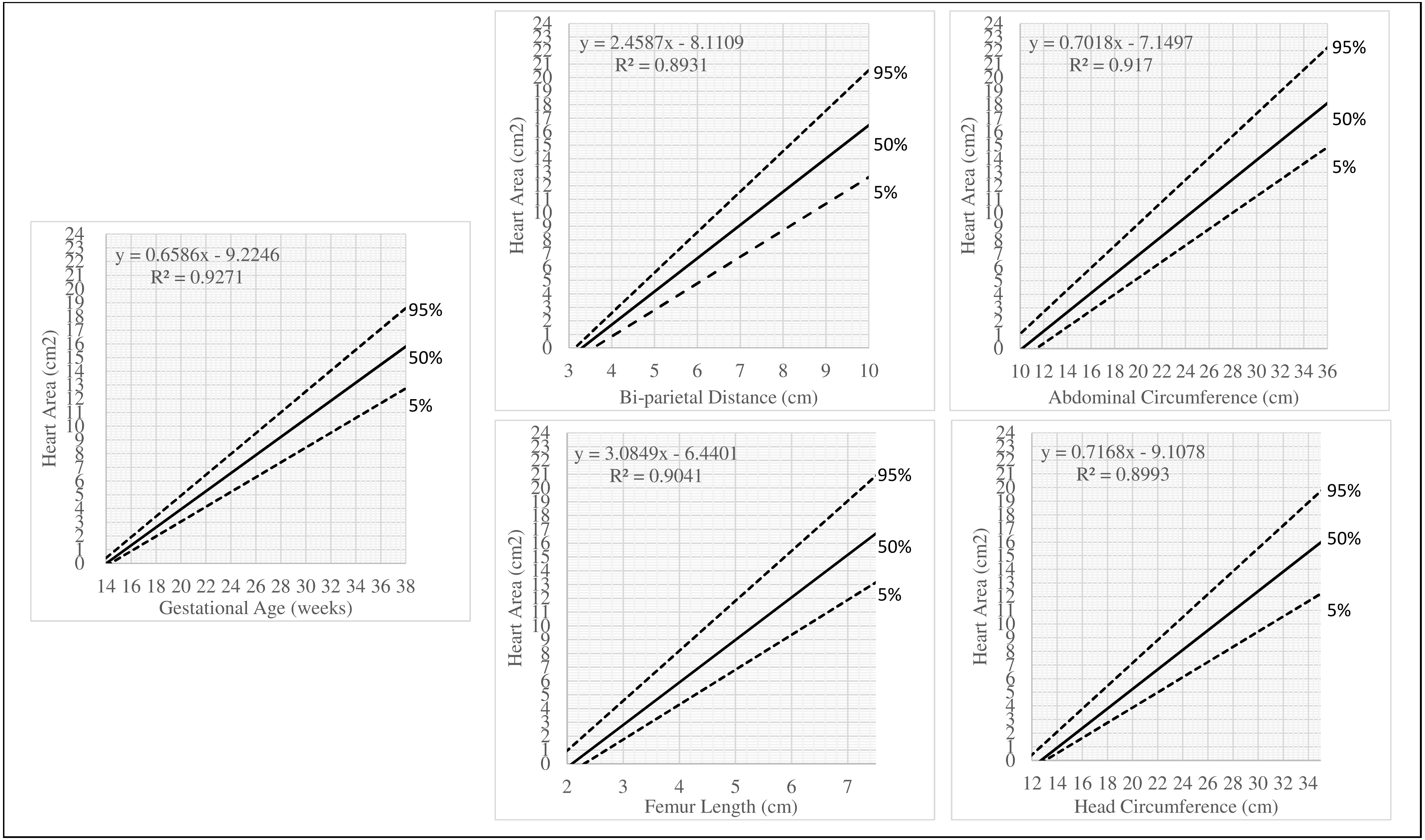

Fig. S1.d. Centile graphs for heart area by estimated gestional age, bi-parietal distance, femur length, abdominal circumference, head circumference.

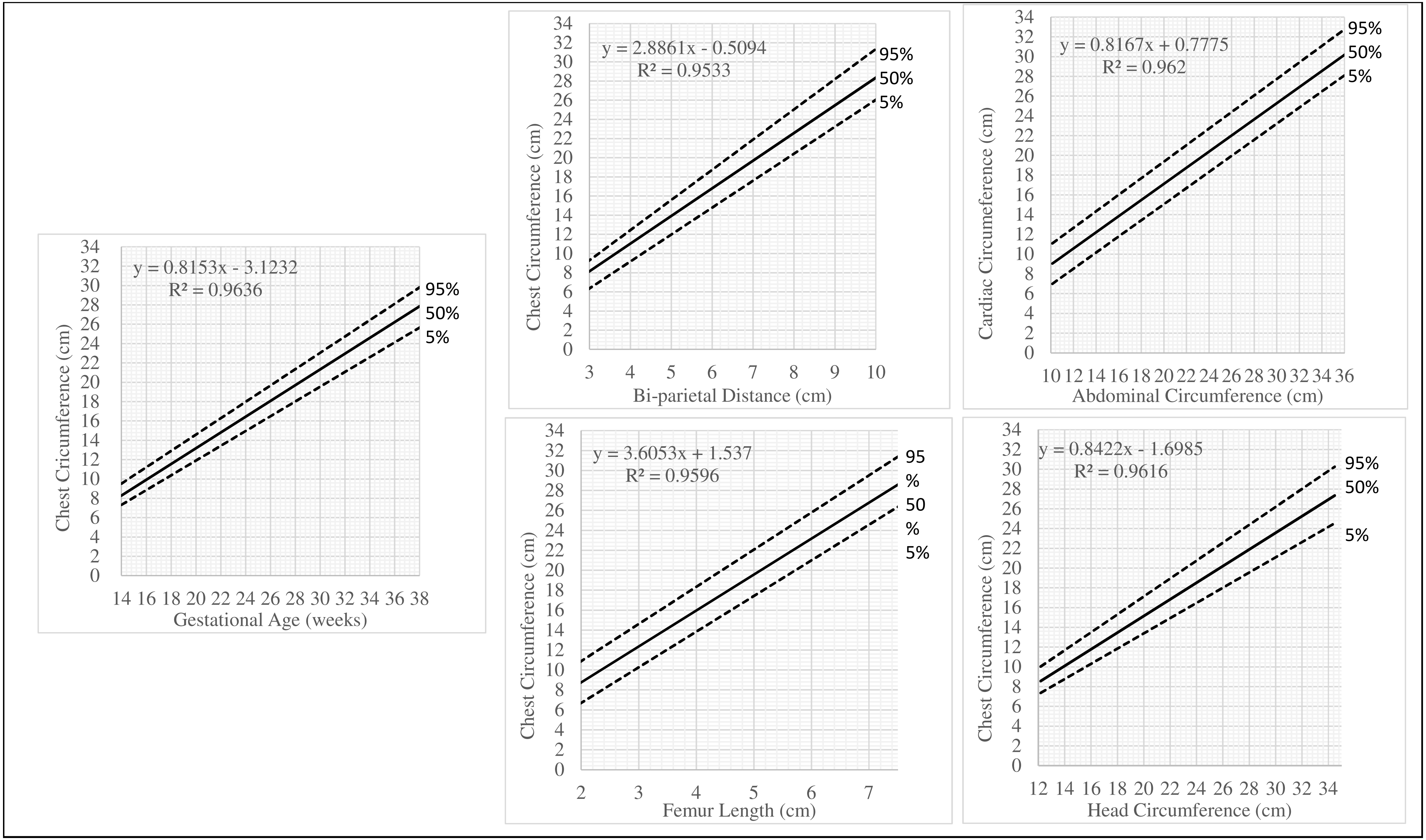

Fig. S1.e. Centile graphs for chest circumference by estimated gestional age, bi-parietal distance, femur length, abdominal circumference, head circumference.

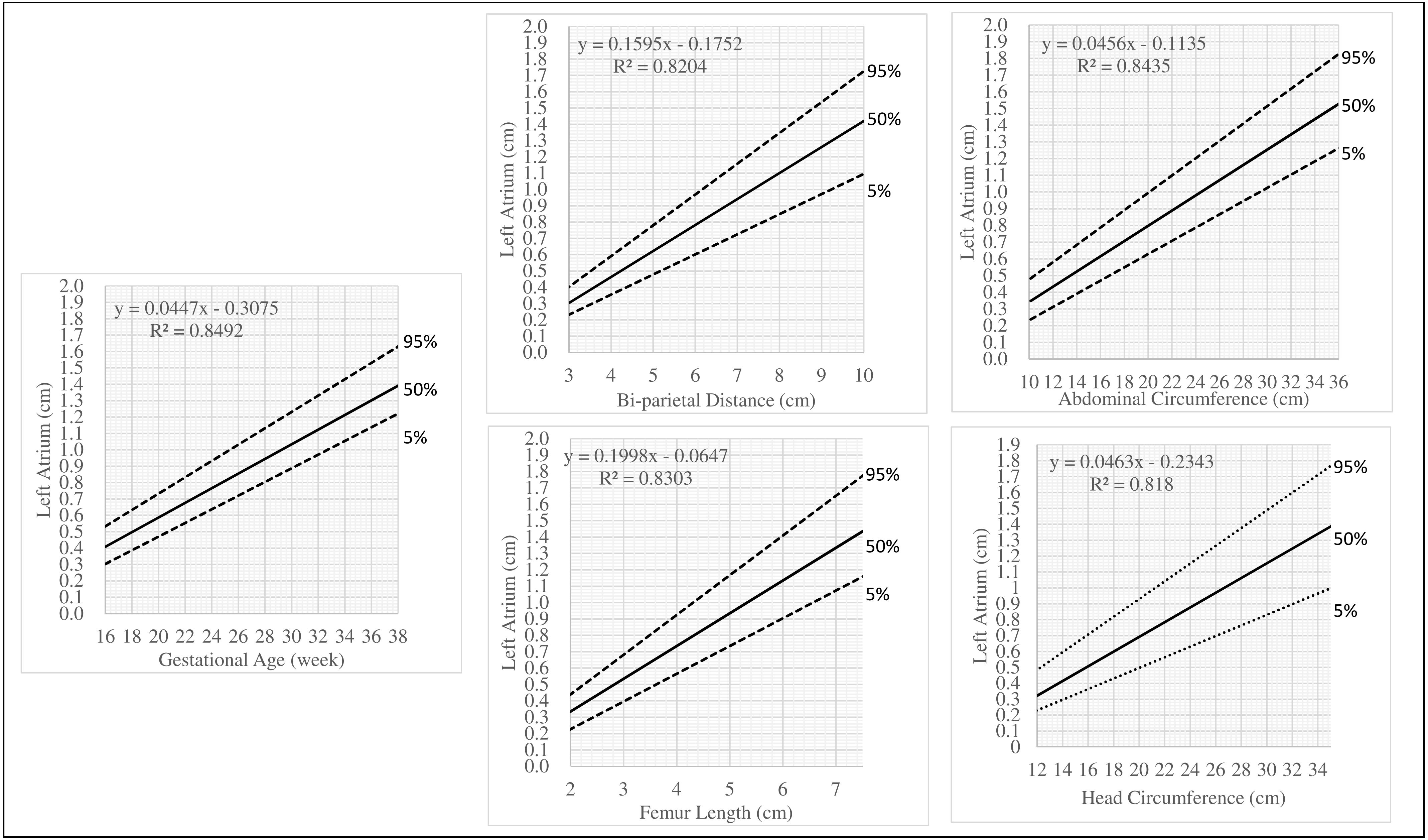

Fig. S1.f. Centile graphs for left atrium by estimated gestional age, bi-parietal distance, femur length, abdominal circumference, head circumference.

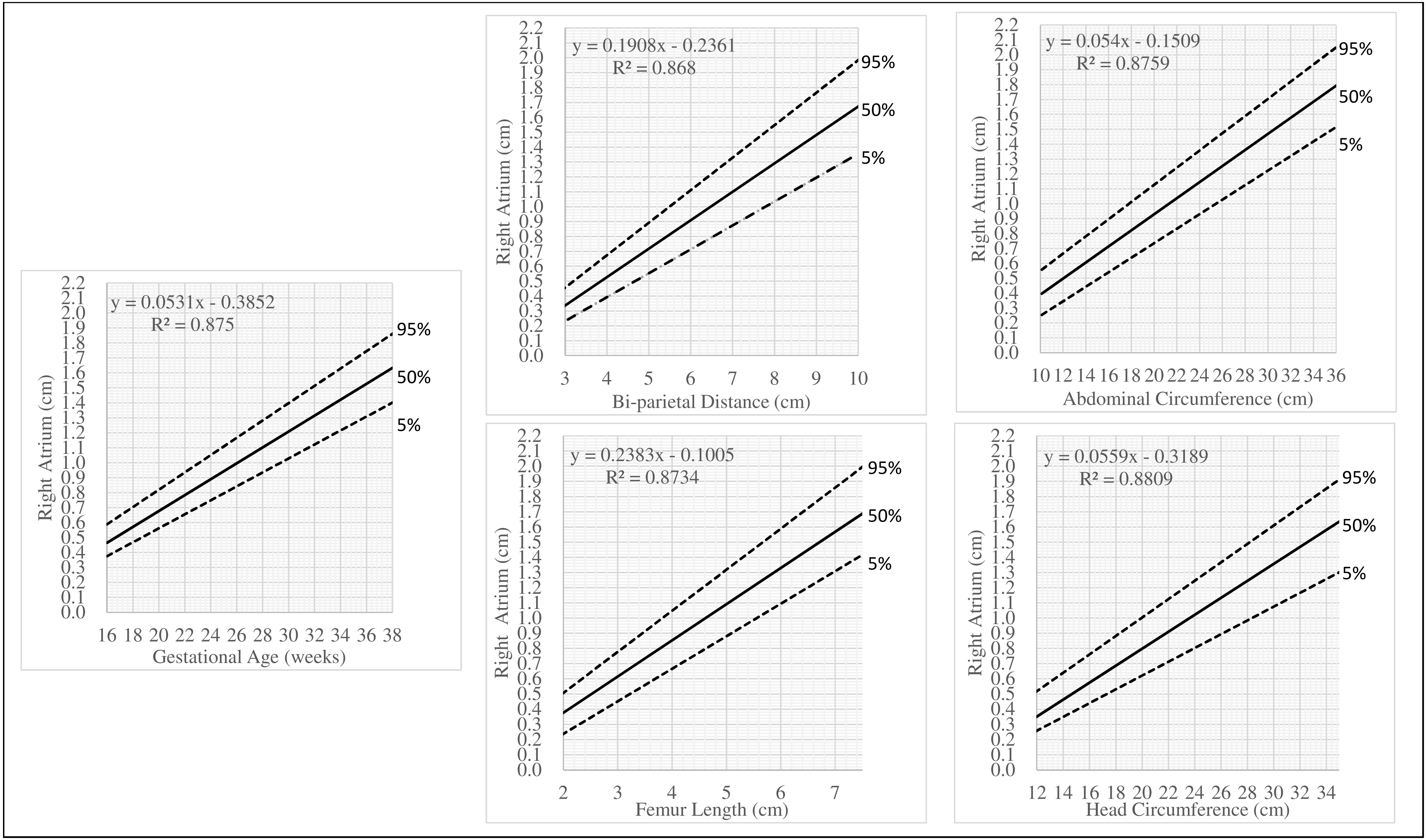

Fig. S1.g. Centile graphs for right atrium by estimated gestional age, bi-parietal distance, femur length, abdominal circumference, head circumference.

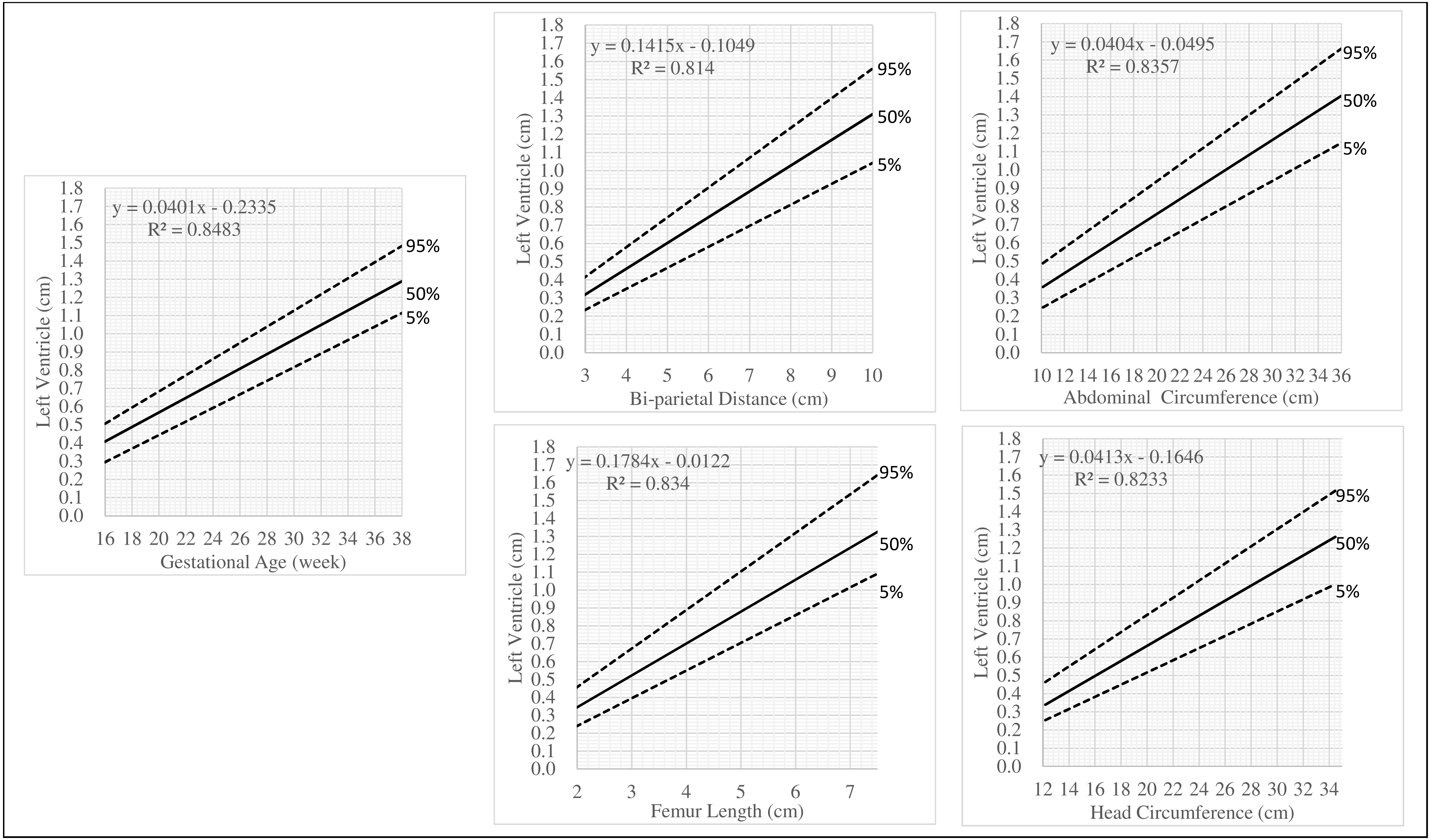

Fig. S1.h. Centile graphs for left ventricle by estimated gestional age, bi-parietal distance, femur length, abdominal circumference, head circumference.

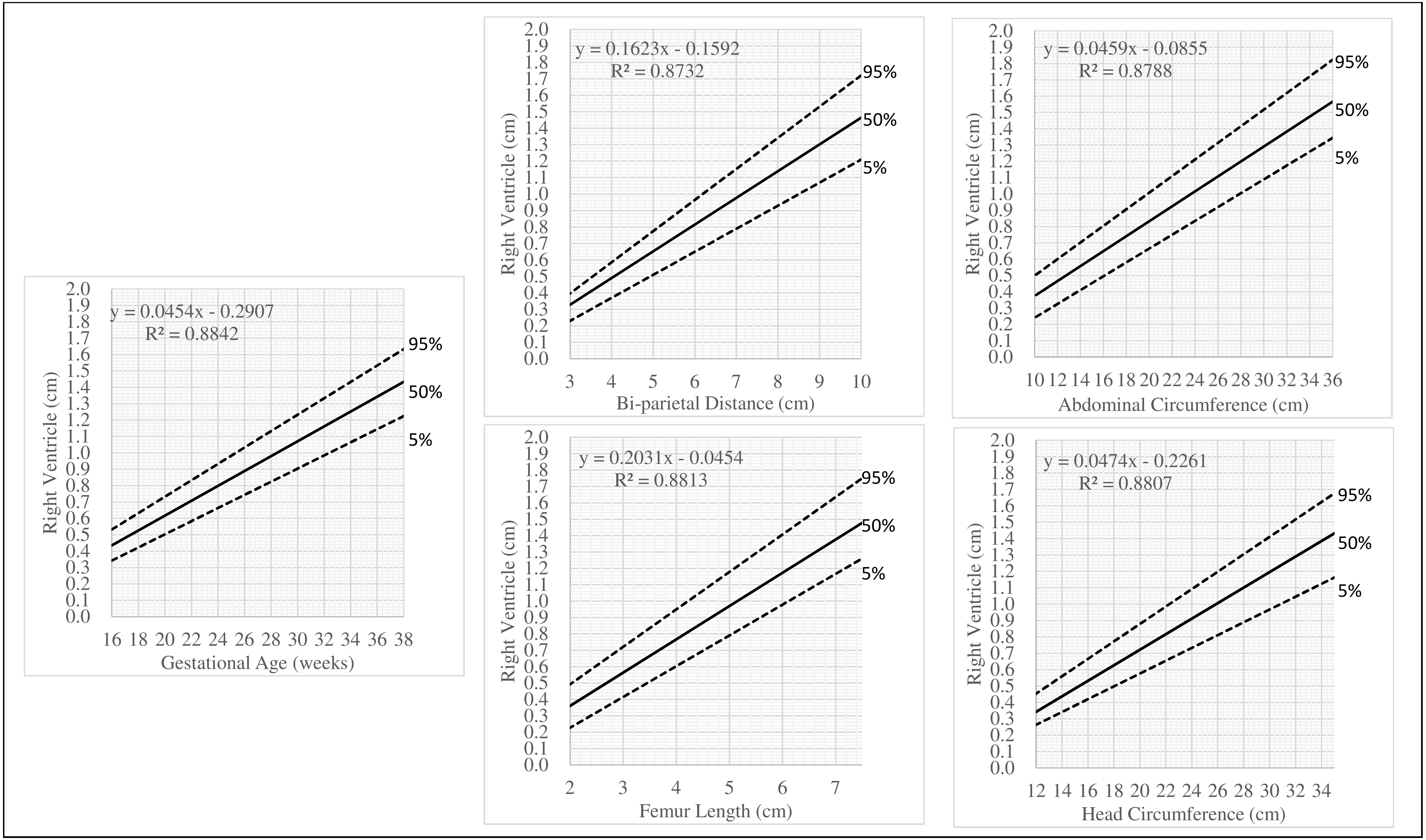

Fig. S1.i. Centile graphs for right ventricle by estimated gestional age, bi-parietal distance, femur length, abdominal circumference, head circumference.

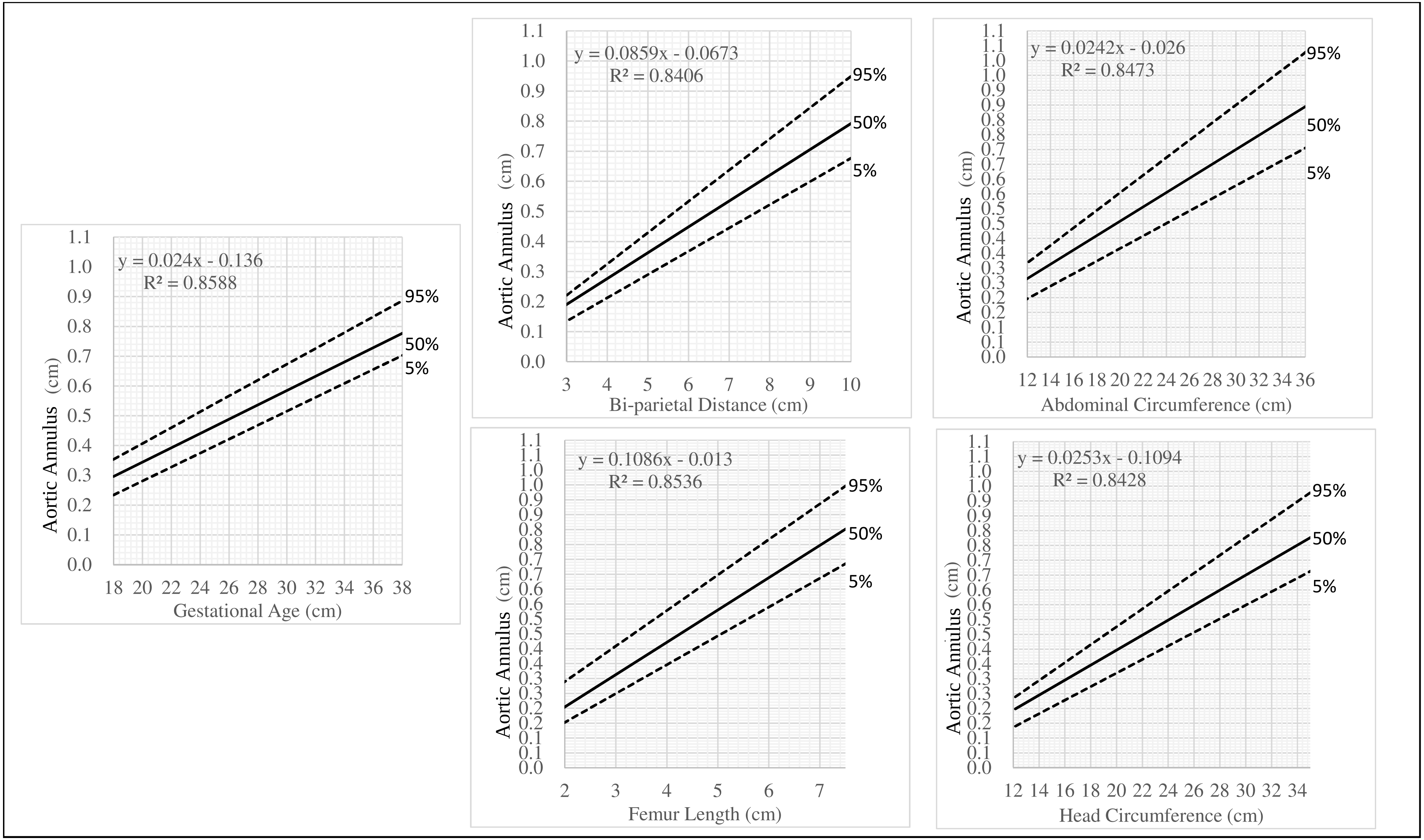

Fig. S1.j. Centile graphs for aorta by estimated gestional age, bi-parietal distance, femur length, abdominal circumference, head circumference.

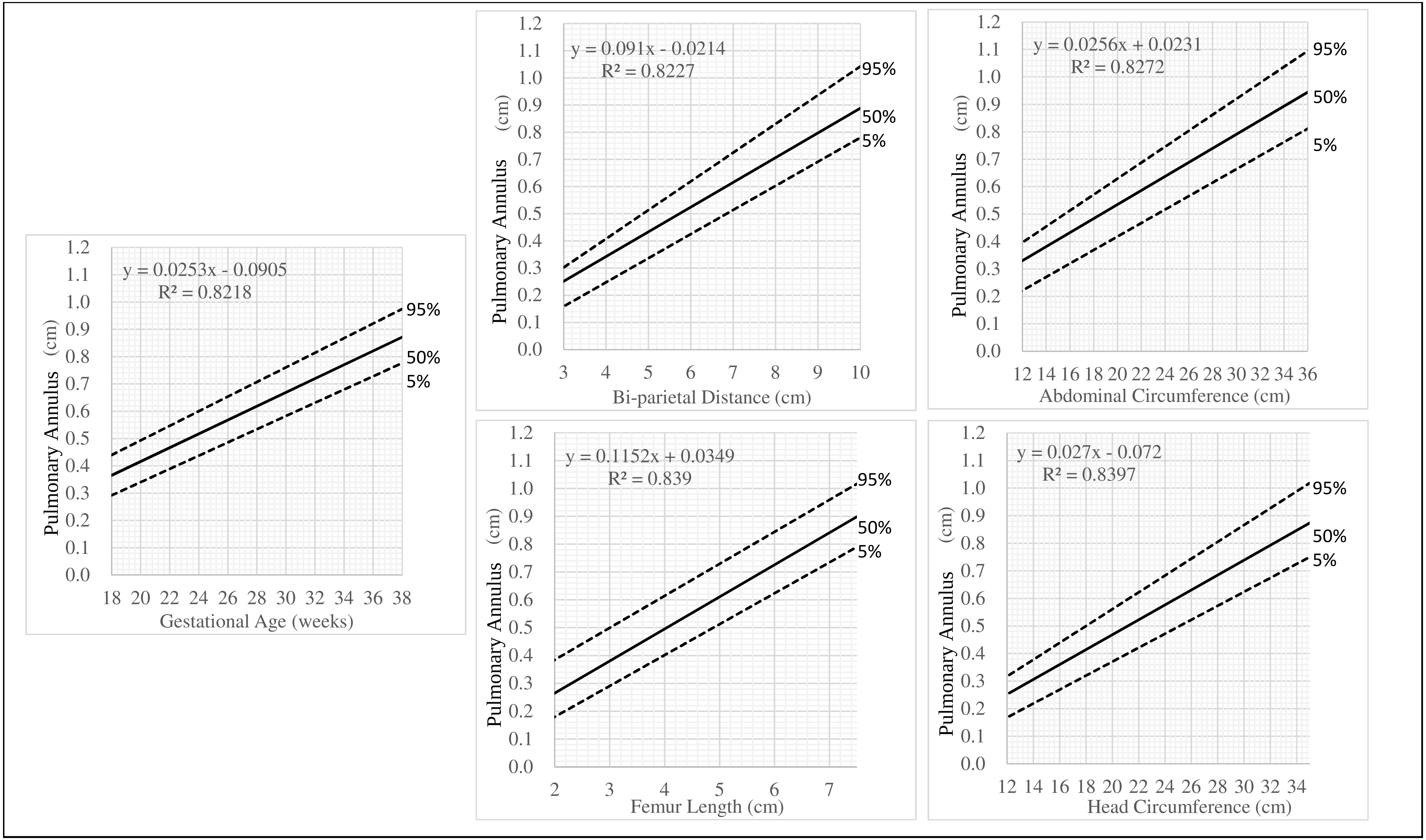

Fig. S1.k. Centile graphs for pumonary artery by estimated gestional age, bi-parietal distance, femur length, abdominal circumference, head circumference.

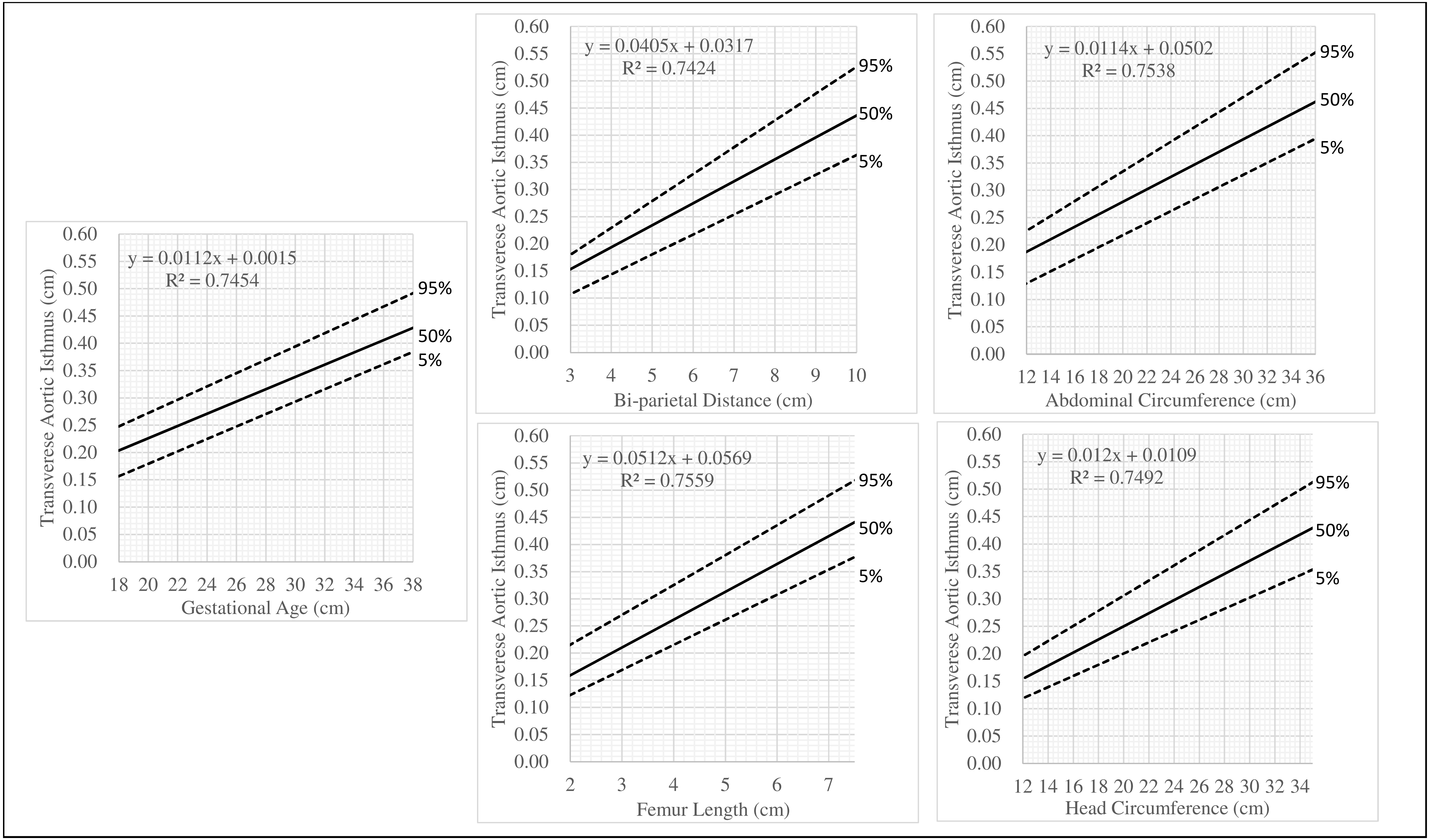

Fig. S1.l. Centile graphs for transverse aortic isthmus by estimated gestional age, bi-parietal distance, femur length, abdominal circumference, head circumference.

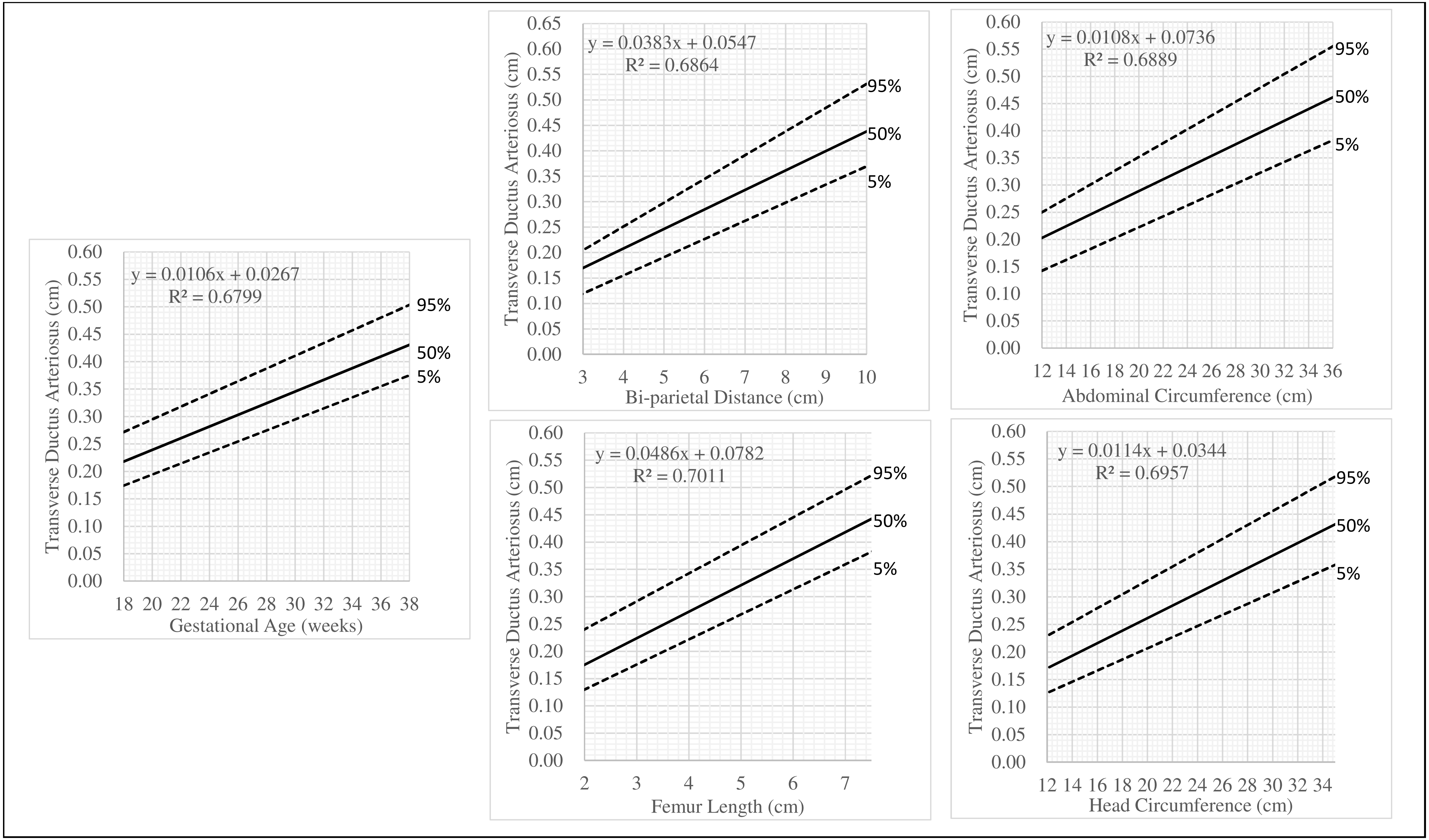

Fig. S1.m. Centile graphs for transverse ductus arteriosus by estimated gestional age, bi-parietal distance, femur length, abdominal circumference, head circumference.
