# Supplementary material for: Reference ranges and Z-scores for fetal cardiac measurements from two-dimensional echocardiography in Asian population
Source: PLoS One. 2020 Jun 25;15(6):e0233179. doi: 10.1371/journal.pone.0233179 (PMC7316227; doi:10.1371/journal.pone.0233179)
Supplement: S2 Fig — a-m. Nomogram for estimated gestational age, bi-parietal distance, femur length, abdominal circumference, head circumference. (PDF) [file pone.0233179.s002.pdf]

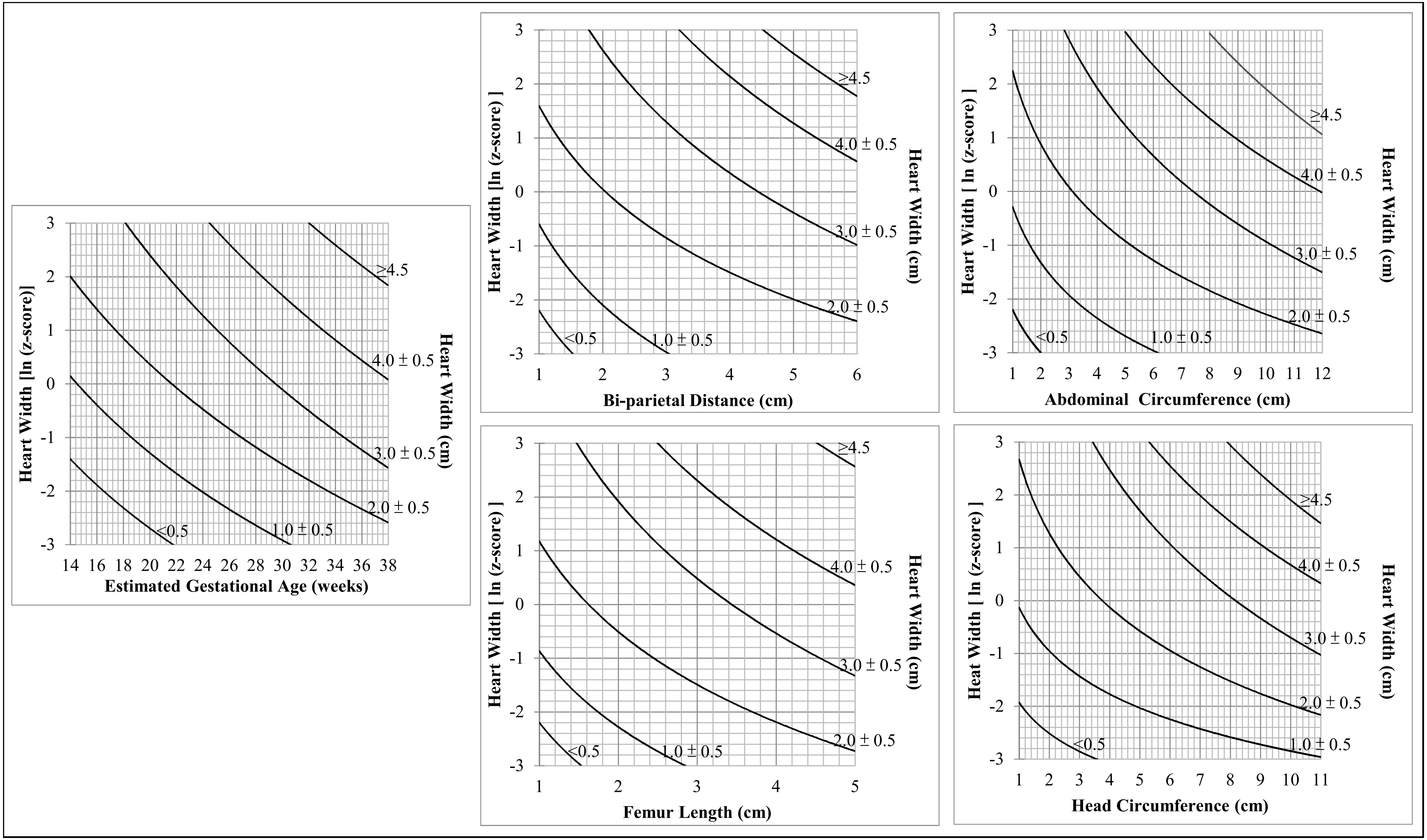

Fig. S2.a. Nomogram for heart width by estimated gestional age, bi-parietal distance, femur length, abdominal circumference, head circumference.

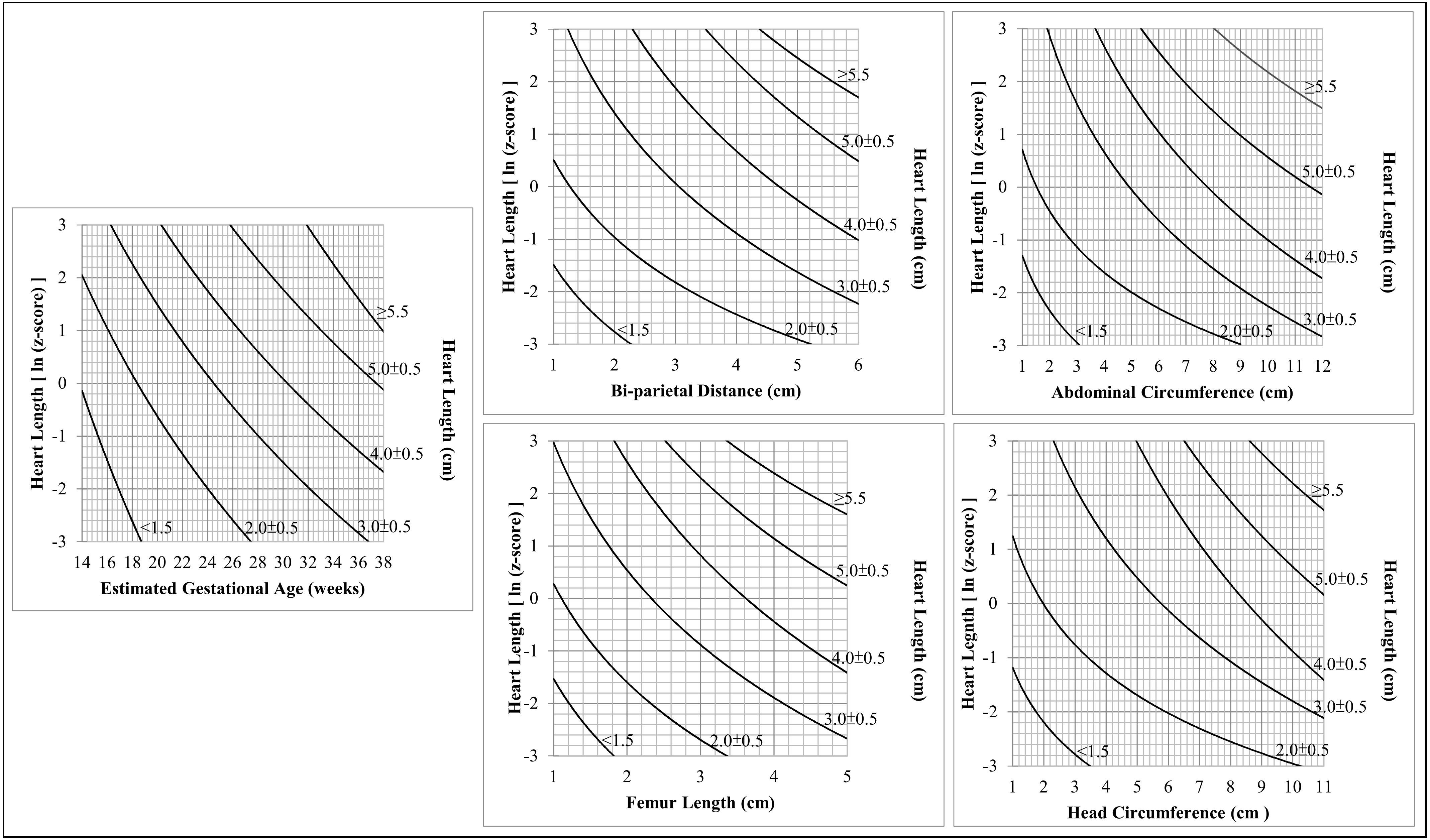

Fig. S2.b. Nomogram for heart length by estimated gestional age, bi-parietal distance, femur length, abdominal circumference, head circumference.

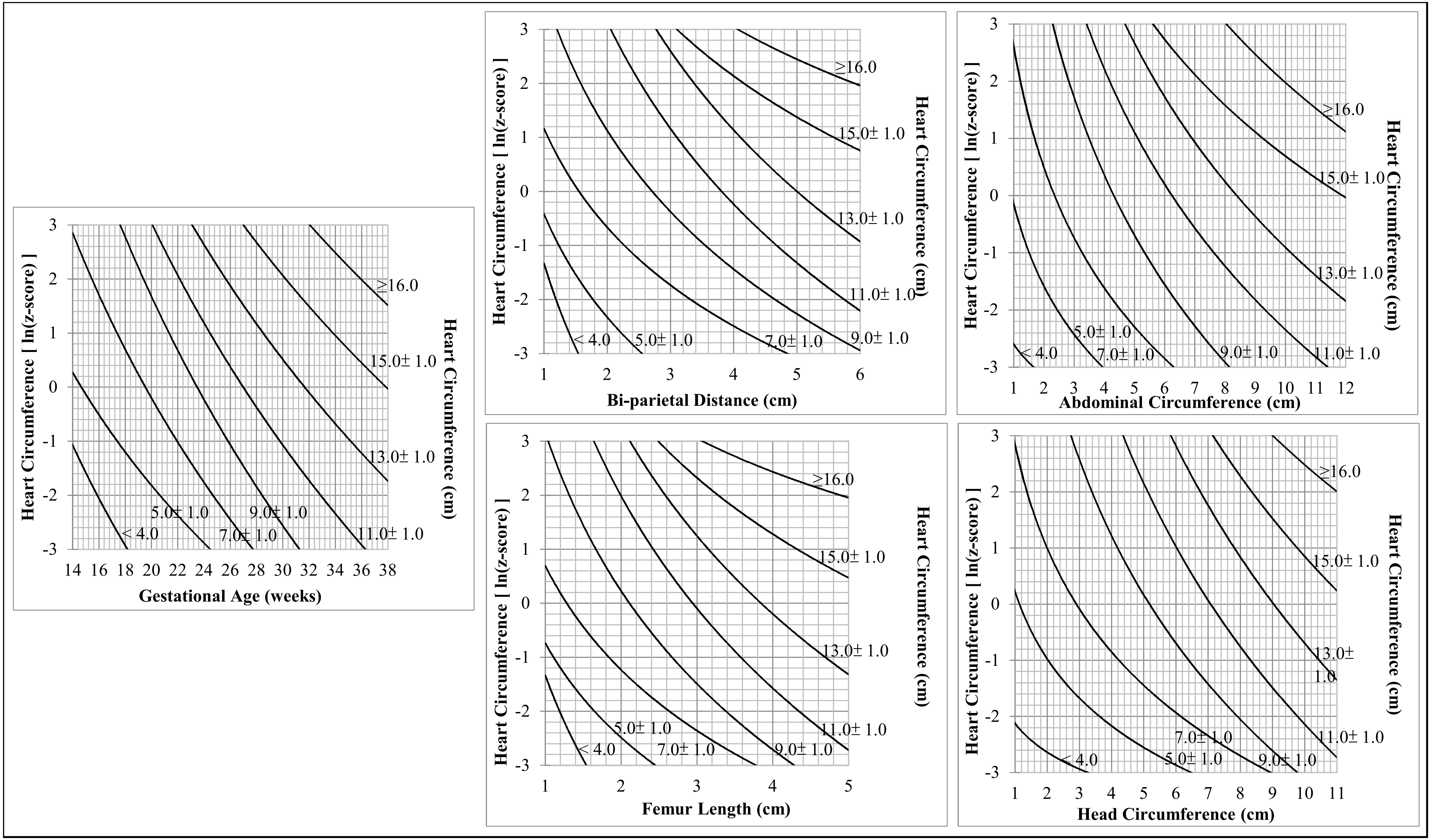

Fig. S2.c. Nomogram for heart circumference by estimated gestional age, bi-parietal distance, femur length, abdominal circumference, head circumference.

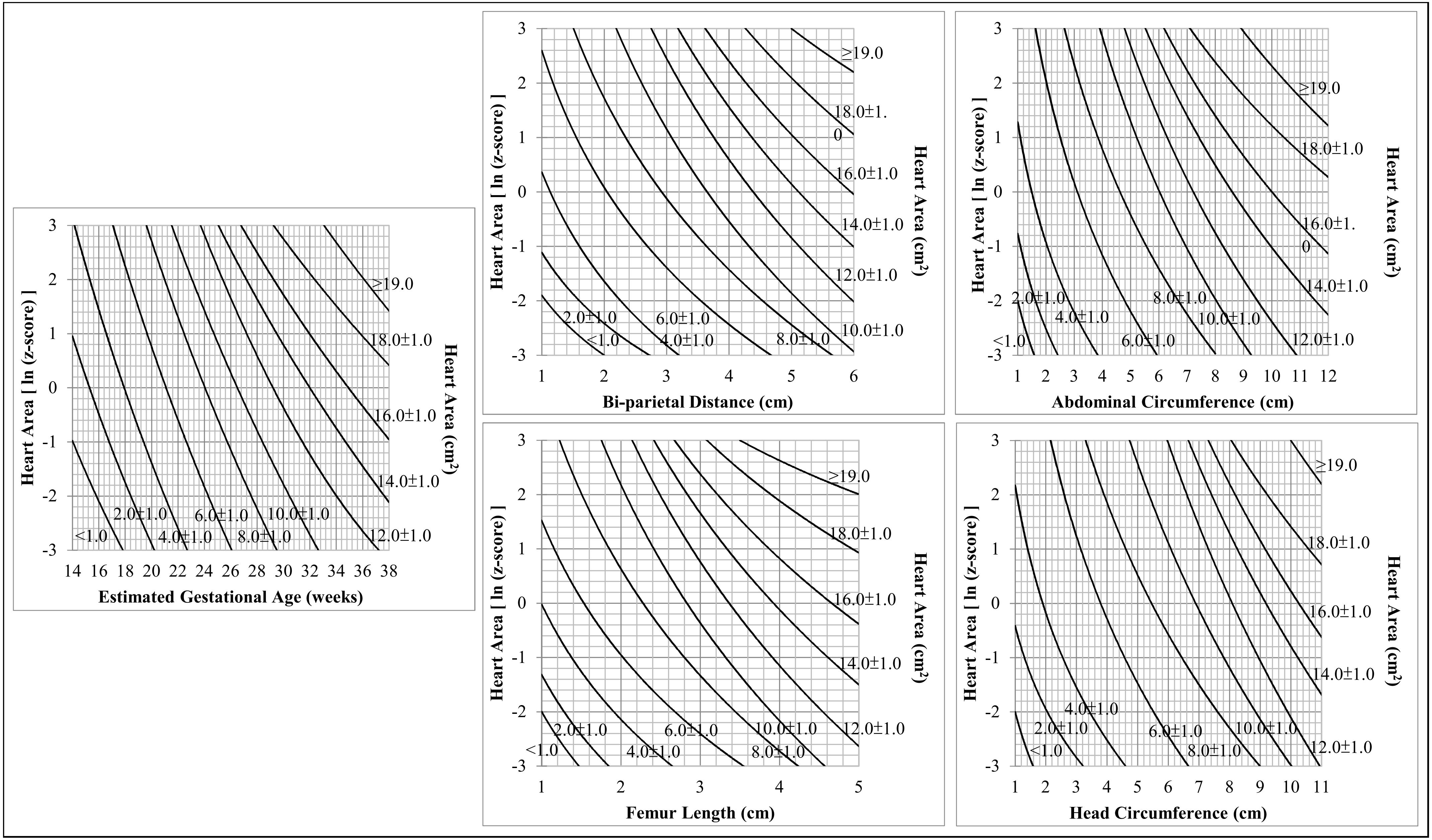

Fig. S2.d. Nomogram for heart area by estimated gestional age, bi-parietal distance, femur length, abdominal circumference, head circumference.

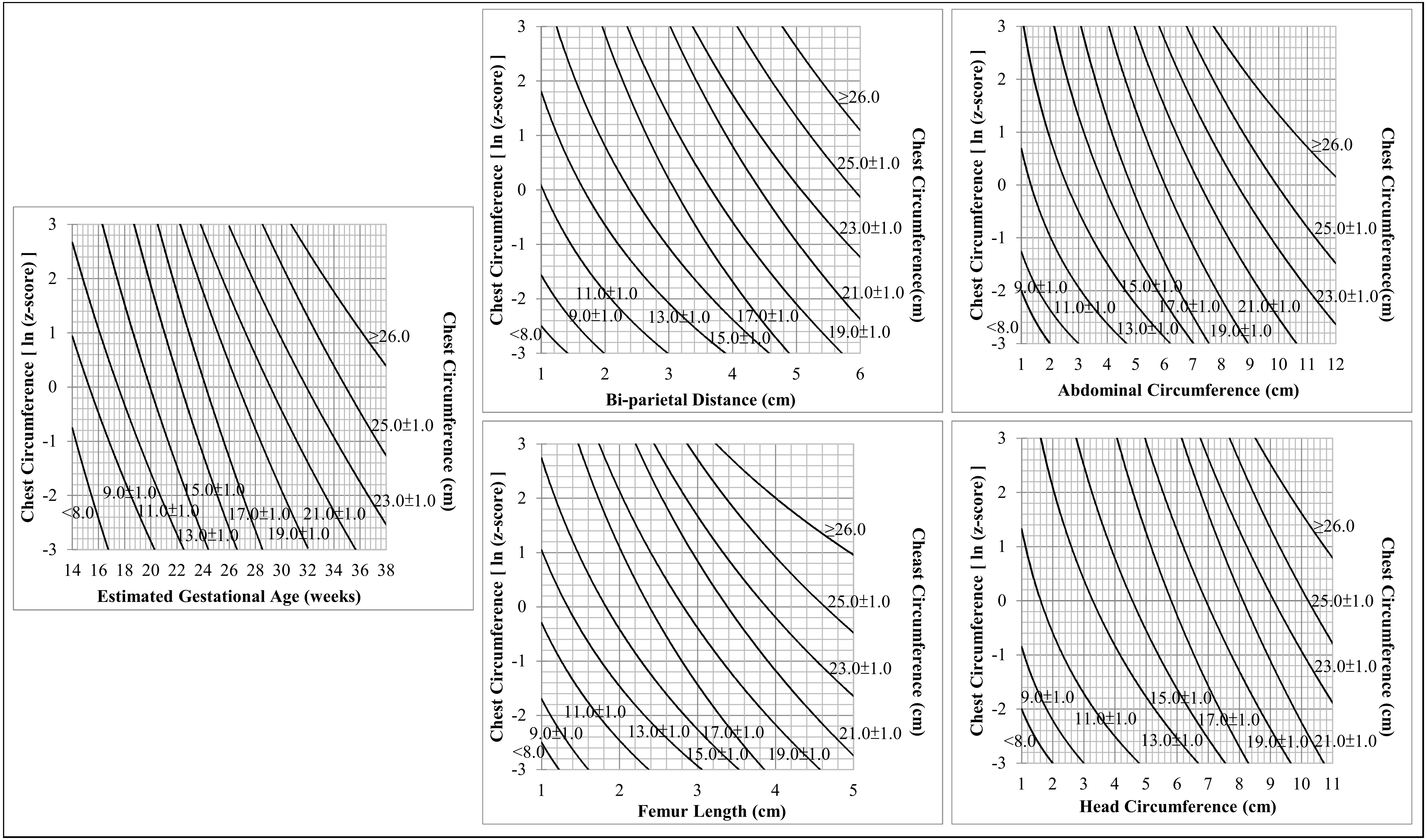

Fig. S2.e. Nomogram for chest circumference by estimated gestional age, bi-parietal distance, femur length, abdominal circumference, head circumference.

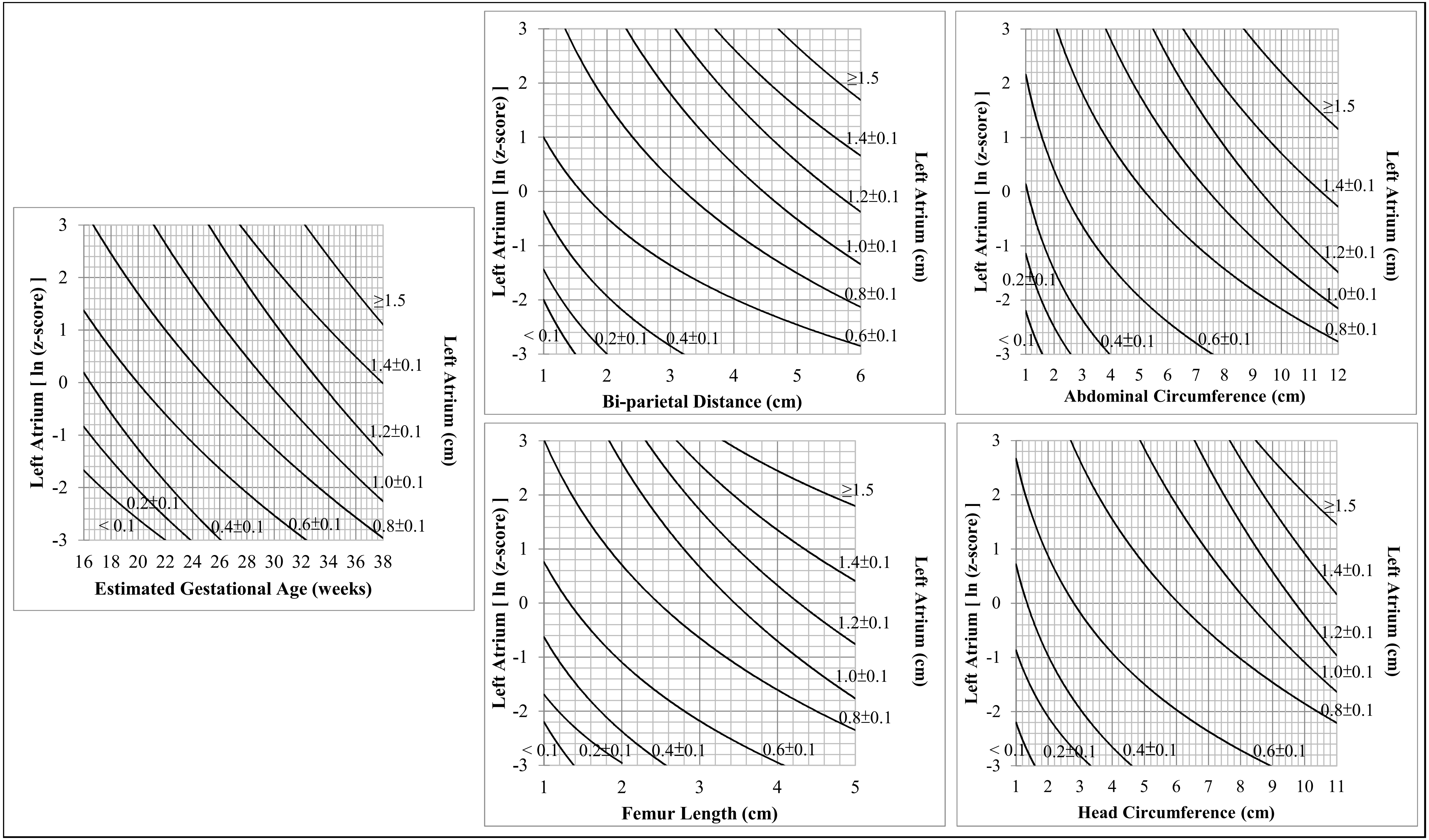

Fig. S2.f. Nomogram for left atrium by estimated gestional age, bi-parietal distance, femur length, abdominal circumference, head circumference.

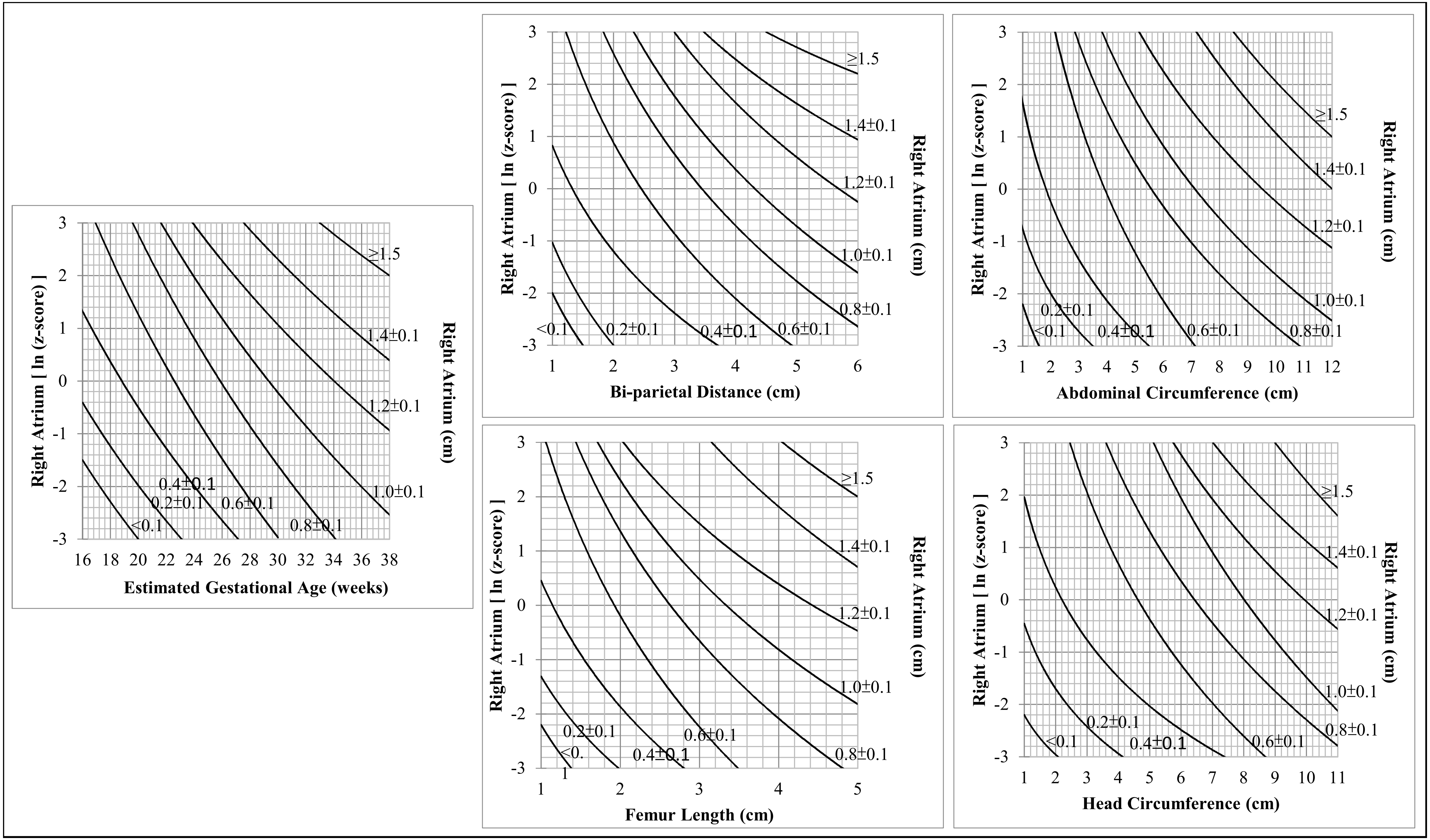

Fig. S2.g. Nomogram for right atrium by estimated gestional age, bi-parietal distance, femur length, abdominal circumference, head circumference.

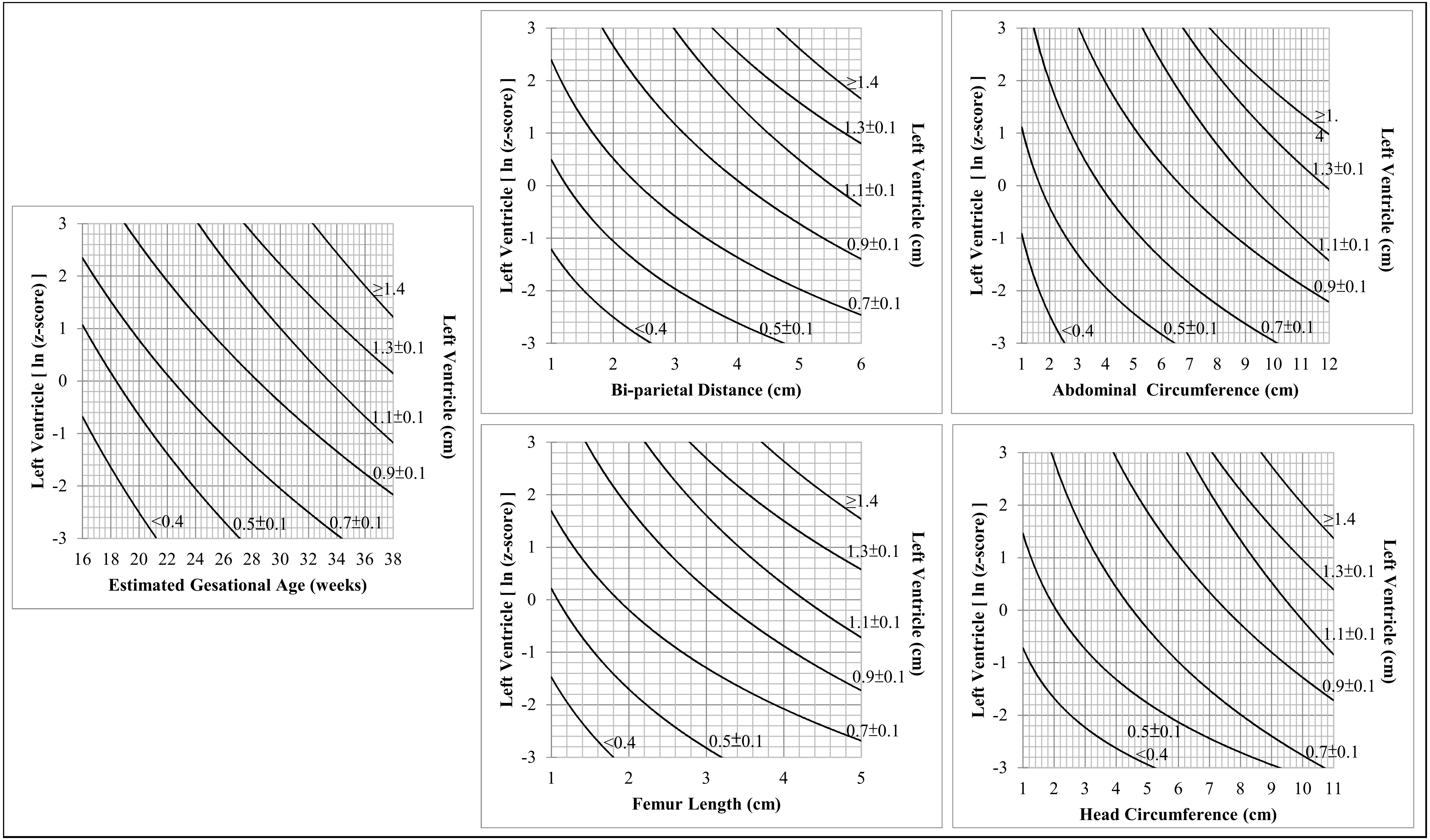

Fig. S2.h. Nomogram for left ventricle by estimated gestional age, bi-parietal distance, femur length, abdominal circumference, head circumference.

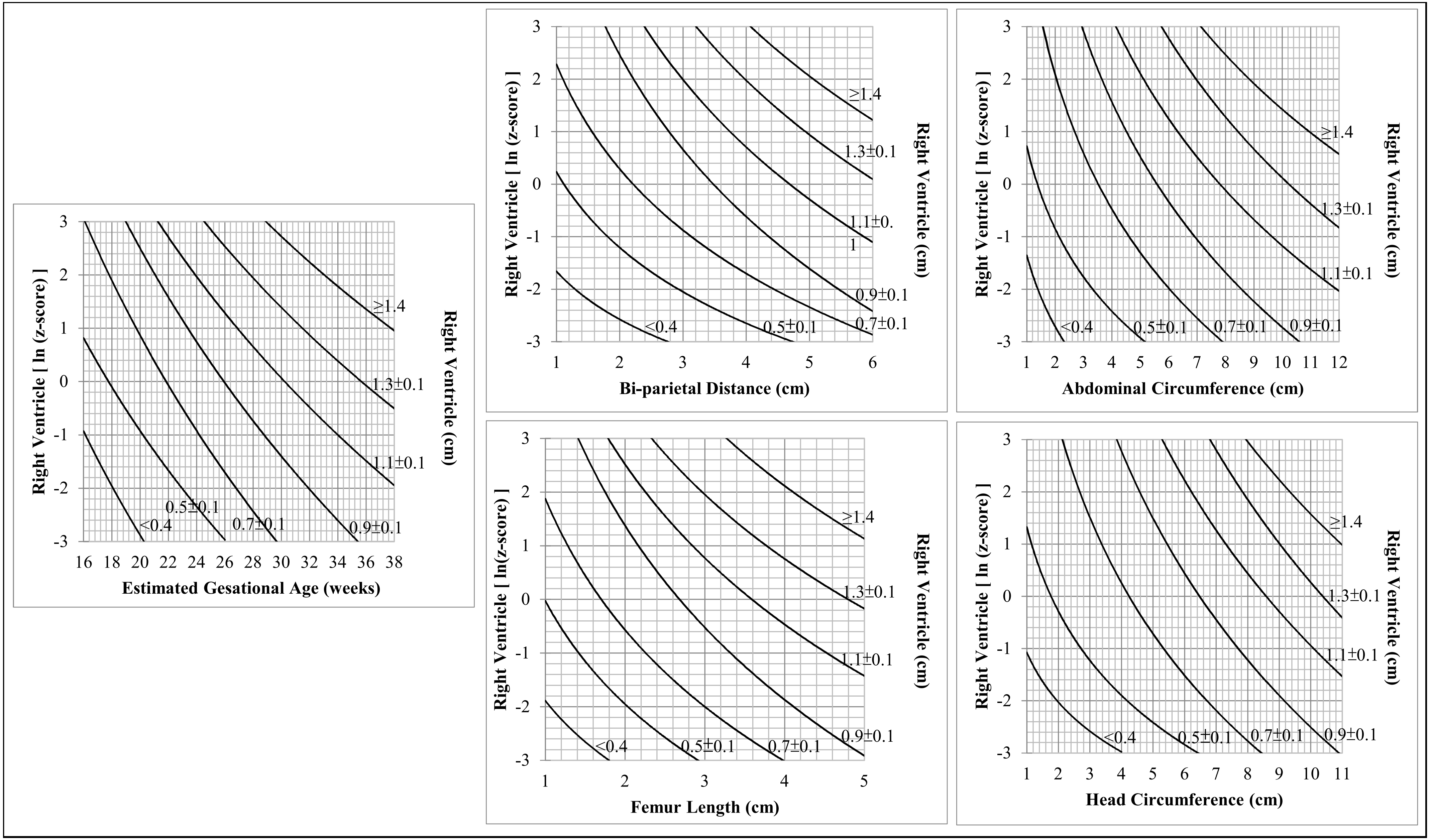

Fig. S2.i. Nomogram for right ventricle by estimated gestional age, bi-parietal distance, femur length, abdominal circumference, head circumference.

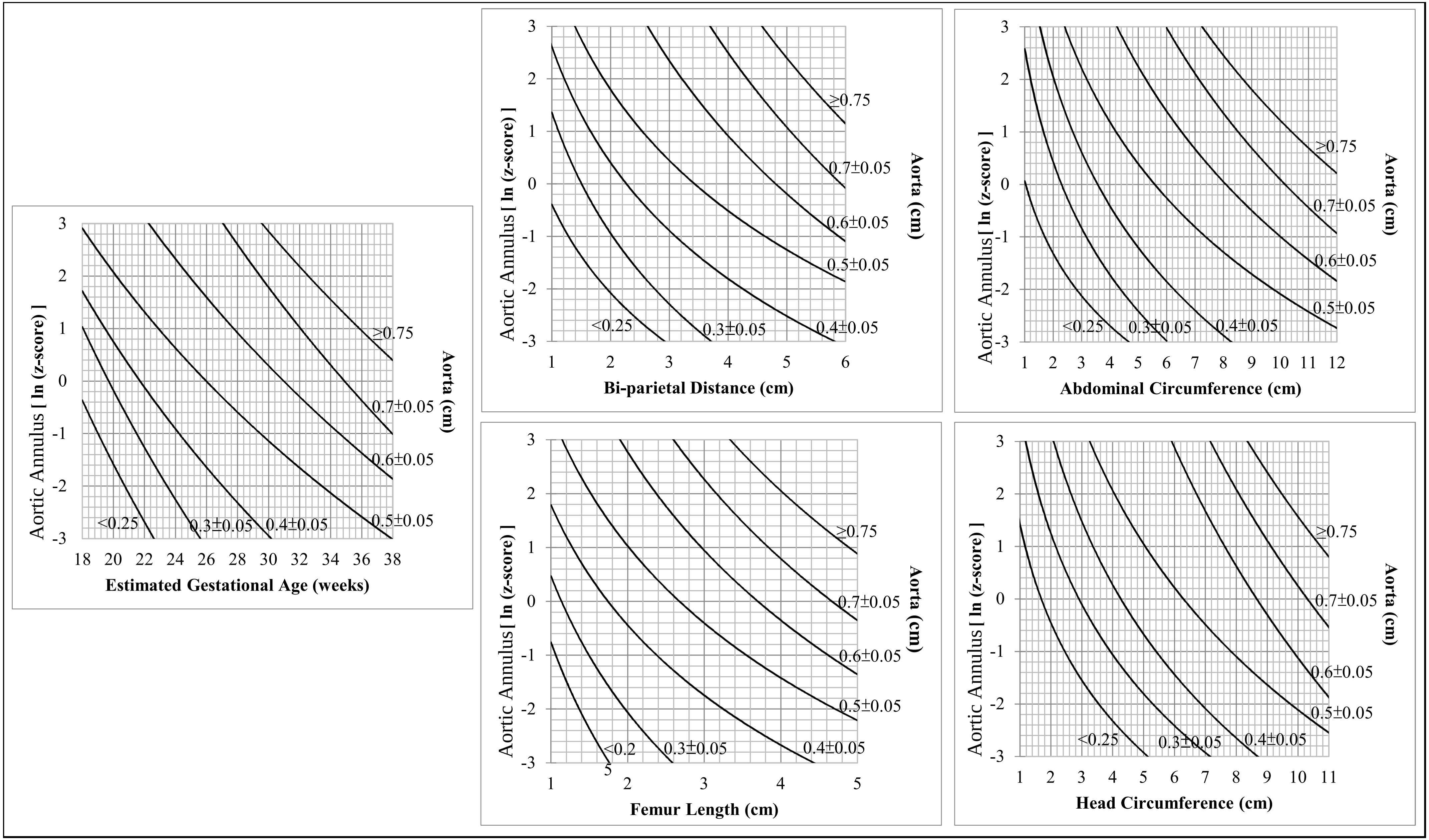

Fig. S2.j. Nomogram for aorta by estimated gestional age, bi-parietal distance, femur length, abdominal circumference, head circumference.

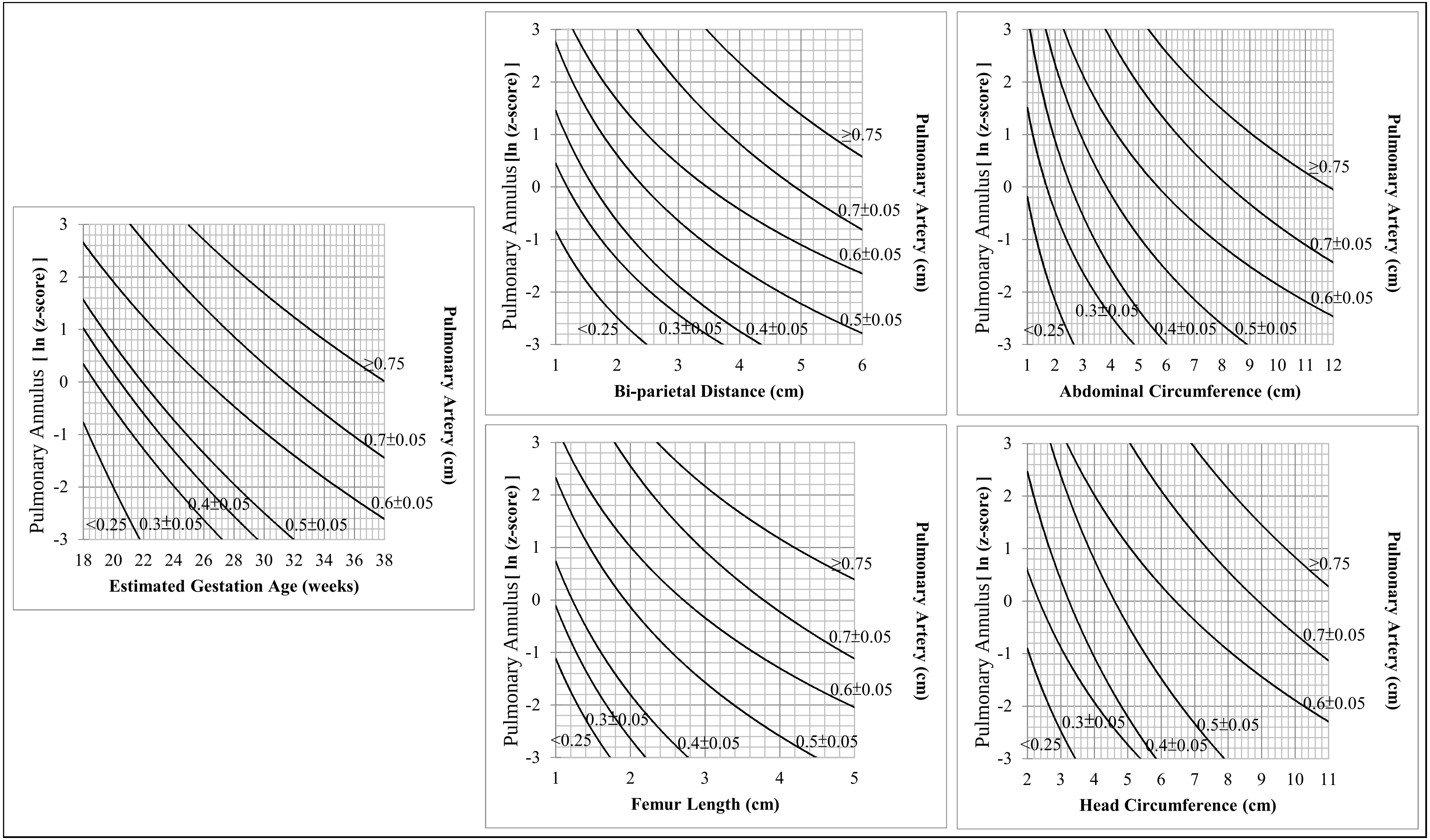

Fig. S2.k. Nomogram for pumonary artery by estimated gestional age, bi-parietal distance, femur length, abdominal circumference, head circumference.

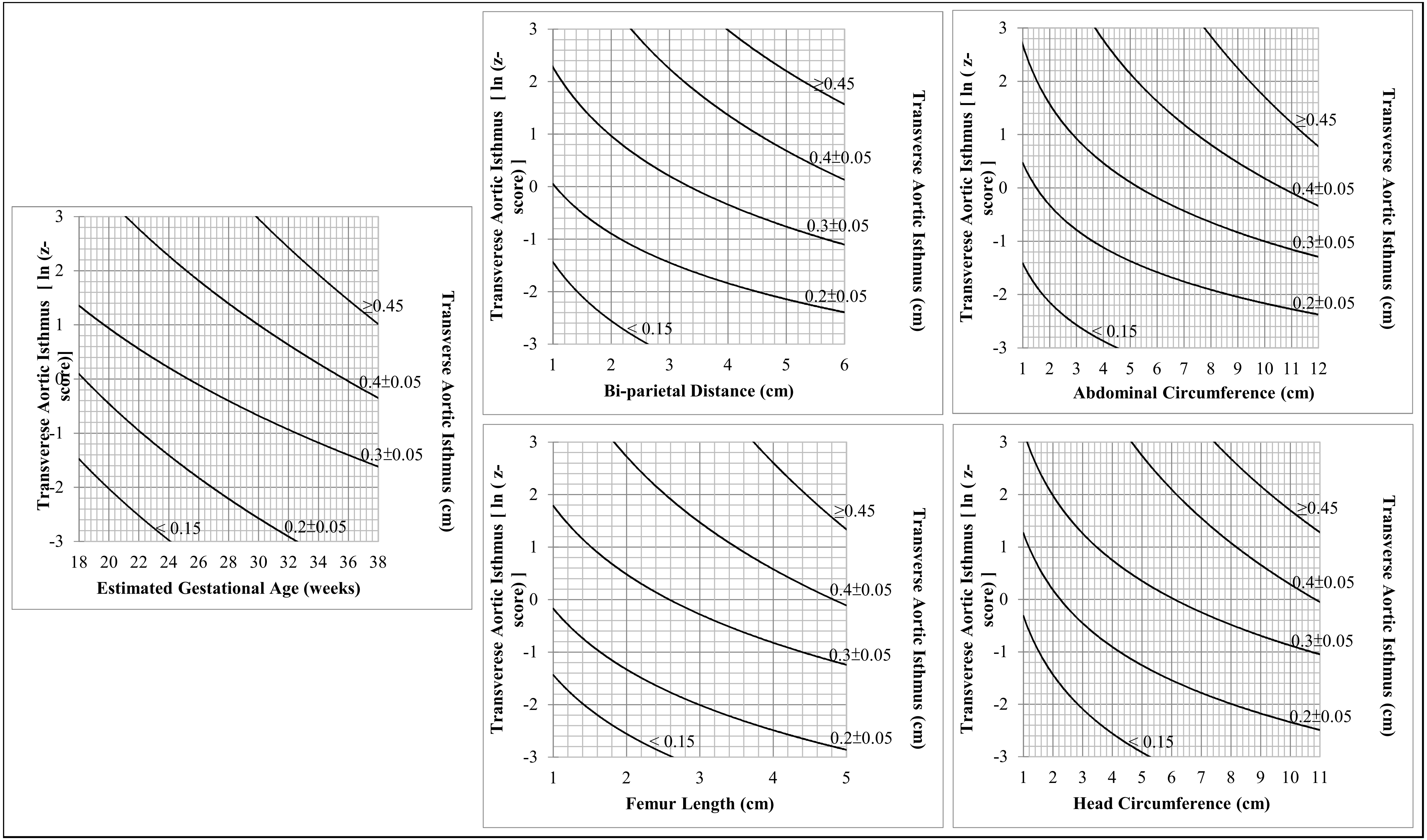

Fig. S2.l. Nomogram for Transverse Aortic Isthmus by estimated gestional age, bi-parietal distance, femur length, abdominal circumference, head circumference.

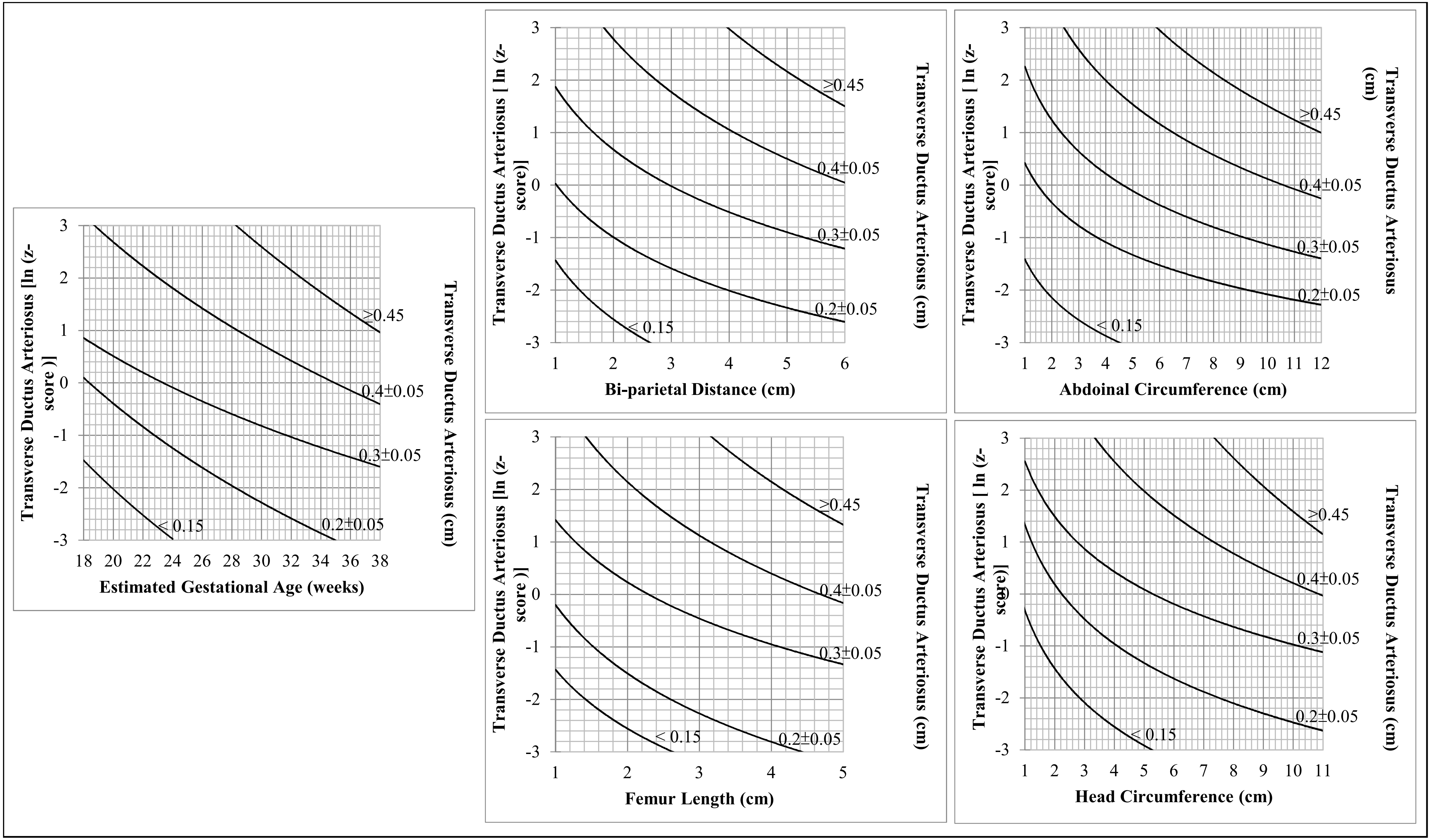

Fig. S2.m. Nomogram for transverse ductus arteriosus by estimated gestional age, bi-parietal distance, femur length, abdominal circumference, head circumference.
