# Supplementary material for: Reference ranges and Z-scores for fetal cardiac measurements from two-dimensional echocardiography in Asian population
Source: PLoS One. 2020 Jun 25;15(6):e0233179. doi: 10.1371/journal.pone.0233179 (PMC7316227; doi:10.1371/journal.pone.0233179)
Supplement: Attachment — Submitted filename: Response To Reviewers.docx [file pone.0233179.s004.docx]

**PONE-D-20-03811**
**Reference ranges and Z-scores for fetal cardiac measurements from two-dimensional echocardiography in Asian population**
*PLOS ONE*

**Responses to the Editors:**

1. **Please ensure that your manuscript meets PLOS ONE's style requirements, including those for file naming. The PLOS ONE style templates can be found at** http://www.plosone.org/attachments/PLOSOne_formatting_sample_main_body.pdf <http://www.plosone.org/attachments/PLOSOne_formatting_sample_title_authors_affiliations.pdf>

*We have made changes to the manuscript to be in line with the PLOS ONE style requirements. The changes made include:*

1. *Reordering line numbering*
2. *Formatting the abstract*
3. *Changing the formatting of headings*
4. *Placing the supplementary captions at the end of the text.*
5. *Reformatting the references*
6. *Reformatting table in text*
2. **Please provide additional details regarding participant consent. In the ethics statement in the Methods and online submission information, please ensure that you have specified (1) whether consent was informed and (2) what type you obtained (for instance, written or verbal, and if verbal, how it was documented and witnessed). If your study included minors, state whether you obtained consent from parents or guardians. If the need for consent was waived by the ethics committee, please include this information.**

*We have updated our consent procedure in the manuscript and have shown that we obtained consent by written approval by participants in the study. A sample of written informed consent is attached below.*

3. **PLOS requires an ORCID iD for the corresponding author in Editorial Manager on papers submitted after December 6th, 2016. Please ensure that you have an ORCID iD and that it is validated in Editorial Manager. To do this, go to ‘Update my Information’ (in the upper left-hand corner of the main menu), and click on the Fetch/Validate link next to the ORCID field. This will take you to the ORCID site and allow you to create a new iD or authenticate a pre-existing iD in Editorial Manager. Please see the following video for instructions on linking an ORCID iD to your Editorial Manager account:** <https://www.youtube.com/watch?v=_xcclfuvtxQ>

*The corresponding author (Chen) has updated their ORCID ID information in the Editorial Manager platform. The ORCID ID number is https://orcid.org/0000-0003-0708-1002*

**Comments to the Author**

**1. Is the manuscript technically sound, and do the data support the conclusions?**

The manuscript must describe a technically sound piece of scientific research with data that supports the conclusions. Experiments must have been conducted rigorously, with appropriate controls, replication, and sample sizes. The conclusions must be drawn appropriately based on the data presented.

Reviewer #1: Yes

Reviewer #2: Partly

2. Has the statistical analysis been performed appropriately and rigorously?

Reviewer #1: Yes

Reviewer #2: Yes

3. Have the authors made all data underlying the findings in their manuscript fully available?

The [PLOS Data policy](http://www.plosone.org/static/policies.action#sharing) requires authors to make all data underlying the findings described in their manuscript fully available without restriction, with rare exception (please refer to the Data Availability Statement in the manuscript PDF file). The data should be provided as part of the manuscript or its supporting information, or deposited to a public repository. For example, in addition to summary statistics, the data points behind means, medians and variance measures should be available. If there are restrictions on publicly sharing data—e.g. participant privacy or use of data from a third party—those must be specified.

Reviewer #1: Yes

Reviewer #2: Yes

4. Is the manuscript presented in an intelligible fashion and written in standard English?

PLOS ONE does not copyedit accepted manuscripts, so the language in submitted articles must be clear, correct, and unambiguous. Any typographical or grammatical errors should be corrected at revision, so please note any specific errors here.

Reviewer #1: Yes

Reviewer #2: Yes

Responses to the Reviewers

**Reviewer #1**: **The article is nice, well written, good statistical analysis. There a few points that merit attention:**

**1) In the method sections and in the tables is not really clear how measurements are made and what they represent (eg. Left atrium, right atrium, right ventricle are not measurements, specify what you measure and insert units, millimeter, cmq or what else). Please specify well. I would add a table on how measurements have been performed**
*Thank you for the suggestion. We have specified how the measurement was made more clearly in Lines 80-95. Units of measurements were explicitly stated as using cm and cm^2^. In summary, we measured heart & chest size, chamber width, aortic annulus (Ao) and pulmonary annulus (PA) in diastole, while measured transverse aortic isthmus (AI) and transverse ductus arteriosus (DA) in its widest systolic diameter. We chose to measure Ao and PA annulus in diastole but not in systole, in order to optimize measurement especially in earlier gestational age. Our measurement dimensions were established based on past studies measuring Ao and PA annulus during diastole (Sharland et al., 1992; Shapiro et al., 1998; Trisha V. Vigneswaran et al., 2018).*

**2) A comparison with other nomograms would be very important. Are the range of normality higher or lower than those previously proposed mainly based on Caucasian population?
In this context discuss and cite also Cantinotti et al. Limitations of Current Fetal Echocardiography Nomograms for 2D Measures: A Critical Overview and Analysis for Future Research. J Am Soc Echocardiogr. 2018 Dec;31(12):1368-1372.e10.**

*Thank you for providing this suggestion and references for support. We have added two new paragraph that add to the discussion section with a comparison between centile graphs and nomograms from our findings to this paper and other previous studies (Lines 247-280).*

**3) These nomograms may be incorporated in current web site providng nomograms (e. parameterz) and mobile App (such as BabyNorm). Please comment and cite at this aim.
*Cantinotti et al. Pediatric echocardiographic nomograms: What has been done and what still needs to be done.Trends Cardiovasc Med. 2017 Jul;27(5):336-349.***

*Thank you for sharing these resources. We have included a section in the conclusion and added the following text to our discussion (Lines 280-286):*

*“We hope to share our best fitting equations and nomograms online on mobile apps and websites that measure fetal echocardiography development (eg. parameterz.com, BabyNorm, etc.)(Cantinotti et al, 2017), to supplement previously developed nomograms and provide novel nomograms for parameters that have not yet been reported. Our measurements could be easily accessible to both patients and physicians alike who are need to compare their measurements among an Asian sample”*

**4) Some helpful measurements are missing such as ventricular thickness. Please add in the limitations.**

*We have added the missing parameters as limitations in our study (Lines 310-312).*

**5) Limitations and Conclusive remarks should be separate by discussion, please add subtitles**

*Thank you to the reviewer for this point. We have added subheadings for Limitation and Conclusion section (Line 288 & Line 314)*.

**Reviewer #2: This is a well-designed prospective study done in a low risk population including almost 600 cases to define normal ranges (Z-scores) of different echocardiographic variables.
The strengths of the paper include:
- reasonable sample size across the range of gestational age.
- clearly defined methodology.
- The authors have used both gestational age and different fetal biometries to base their normal ranges on.
-Extensive documentation is provided with graphs representing 5th and 95th percentiles

However, several issues should be addressed:**

**Abstract:**

**Results section in the abstract should be reduced**

*Thank you to the reviewer for his feedback on the abstract. We have shortened the abstract, especially the results section.*

**Methodology:**
**• No baseline characteristics are included such as use of assisted reproductive techniques or maternal diabetes which may have an impact on the fetal heart (Valenzuela-Alcaraz B, Circulation 2013; Patey O, UOG 2019).**

*We retrospectively excluded cases with any maternal disease diagnosed during pregnancy, including GDM and cases of growth restriction in later pregnancy or small for gestational age at birth. The proportion of ART cases in our sample is 4%, which is similar to general population in Taiwan (4.3%) (Hsu JC, Su YC, Tang BY, Lu CY). Use of assisted reproductive technologies before and after the Artificial Reproduction Act in Taiwan. PloS one. 2018;13(11):e0206208). We believe our sample may represent the heterogenicity of fetal heart growth in Asian fetus without growth restriction. However, for clarity we have added a new paragraph in the limitation section illustrating the issue of including ART cases in our sample (Lines 303-310).*

**• No comprehensive fetal assessment was performed including estimated fetal weight assessment. Fetal growth restricted fetuses (under the 10th percentile) shouldn’t be included in nomograms’ construction as they may present cardiac remodelling (Rodríguez-López M, UOG 2017).**

*We prospectively excluded any fetuses that had FGR so they would not have impacted our sample, and only represented normally developed fetuses. We have clarified the inclusion & exclusion criteria in method section and added reference of the fetal biometry we used (Line 56-57).*

**• Atrial measurement should be performed at its maximal distension (end-systole) JS Carvalho UOG 2013.**
*Thank you for feedback on this point. Atrial size in end-systole represents the largest diameter of atrium in cardiac cycle. However, we chose to measure in diastole, which is the same as Shapiro et al., 1998, and also hope to provide a reference with simple measuring process.*

**No inter or intraobserver variability was studied.**

*This is indeed a limitation of our study, since we only had one sonographer, interobserver variability was not studied. However, to ensure quality of measurement and assessment, all measurements were confirmed by an obstetrician-gynecologist and a pediatric cardiologist during patient visits. We have explained this in both method and limitation sections (Line 76-78 & line 295-300).*

**• There is no data available on the feasibility of the studied parameters.**
*Thank you for the feedback. We have added reference 14-22 in the introduction section to justify the description about feasibility of the studied parameters. (Line 39-42):” In clinical practice, z-scores references are practical not only in the screening and diagnosis of fetal cardiac structural abnormalities (reference 14-18), but fetal cardiologist also use z-scores to predict and counsel about possible postnatal outcome and treatment strategies (reference 19-22).”*

• **Discussion** **should be reduced.**

*Thank you for your feedback on the discussion section. We have shortened the discussion section for parts that were repeating and have rearranged some sections for clarity and conciseness.*

**• There is no consistent data to state that “For cases with uncertain gestational age, head circumference could be used as an optimal developmental marker to predict fetal heart growth” in line 347-349**

*We have revised this statement to now state, “For certain fetal heart structures head circumferences can be used as a developmental marker to aid in predicting fetal heart growth.” (Line 245-246)*
